# Zircon record of an Archaean crustal fragment and supercontinent amalgamation in quaternary back-arc volcanic rocks

**DOI:** 10.1038/s41598-021-90578-9

**Published:** 2021-06-11

**Authors:** Zhi-gang Zeng, Zu-xing Chen, Yu-xiang Zhang

**Affiliations:** 1 Key Laboratory of Marine Geology and Environment, Institute of Oceanology, Chinese Academy of Sciences, Qingdao, 266071 China; 2 Laboratory for Marine Mineral Resources, Qingdao National Laboratory for Marine Science and Technology, Qingdao, 266071 China; 3 Center for Ocean Mega-Science, Chinese Academy of Sciences, Qingdao, 266071 China; 4 University of Chinese Academy of Sciences, Beijing, 100049 China

**Keywords:** Geochemistry, Geodynamics, Geology, Mineralogy, Petrology, Tectonics, Volcanology

## Abstract

Magmatism has profoundly influenced the evolution of the geosphere, hydrosphere, atmosphere, and biosphere in back-arc basins. However, the timing of the magmatism in the Okinawa Trough (OT) is not well constrained by the age spectra of zircons. Here, for the first time, we carry out an integrated study combining in situ analysis of zircon U–Th–Pb and Hf–O isotopes, and trace element compositions of zircons from the volcanic rocks from the southernmost part of the OT. We found that the young (< 100 ka) zircons in these volcanic rocks have old (108 Ma to 2.7 Ga) inherited cores, which were captured as the magma ascended through the rifting continental crust. In particular, the inherited Archean zircons strongly suggest that remnants of the old East Asian continental blocks underlie the embryonic crustal rifting zone. Moreover, the ages of most of the inherited zircons correspond to five supercontinent amalgamation events. Specifically, the Archaean inherited zircons, which have positive εHf_(t)_ and low δ^18^O values, correspond to the formation of juvenile continental crust. In contrast, the negative εHf_(t)_ and high δ^18^O values of the post-Archaean inherited zircons indicate that their parental magma contained recycled older crust due to the enhanced crust-mantle interactions during the evolution of the early continental crust. Therefore, the inherited zircons in the back-arc volcanic rocks not only reflect the evolution of the local magmatism, but they also contain a record of the Archaean crustal fragment and of several global continental amalgamation events.

## Introduction

The OT, which is located on the western Pacific active continental margin (Fig. S1), is a back-arc basin formed by the northwestward subduction of the Philippine Sea Plate beneath the Eurasian Plate, which initiated during the Middle to Late Miocene (i.e., from > 15 Ma to ~ 6 Ma)^1^. However, the nature of the basement is a controversial issue. Conventional wisdom suggests that the OT basement may be an offshore part of the continental lithosphere of the South China Block, which was related to the northwestward subduction of the Philippine Sea Plate beneath the Eurasian Plate^1,2^; In contrast, an allochthonous origin for the OT basement has been proposed by Niu et al.^3^, that is, it was originally a thinned continental block within the palaeo-Pacific Plate and moved westward with the palaeo-Pacific Plate. However, the lack of basement rock samples has limited our understanding for these issues. The crustal thickness decreases from > 25 km in the northern OT to ~ 10 km on the axis of the SOT graben^2^. The southernmost part of the OT (SPOT) is considered to be an embryonic crustal rifting zone^4^, in which the crust (25–30 km) has not experienced significant thinning^4,5^ and is characterized by a cluster of active volcanoes dominated by dacites and rhyolites^4–7^. These < 0.2 Ma silicic magmas evolved via mixing of a mantle-derived basaltic magma and a crustal felsic magma, followed by extensive fractional crystallization^5,6^. Mg element diffusion chronology of the plagioclase in the dacites suggests that relatively long-lived magma storage (at least 600 yrs) occurred in the shallow silicic magma reservoir^5^. However, the duration of the submarine volcanism in the OT is still poorly constrained. At present, no studies have reported the timing of the magmatism based on age spectra of zircons from the OT volcanic eruptions.

Zircon, which is a common accessory mineral, is a powerful tool for understanding magmatic systems because it often records the protracted history prior to eruption^8–11^. Furthermore, inherited zircons in volcanic rocks can provide samples of deeper crustal levels, which makes them a powerful tool for unlocking the mysteries of the Earth’s deep crust^12–15^. In this study, we used Quaternary magmatic zircons from the SPOT volcanic rocks and old inherited zircon cores captured from the deep crust during mantle-derived magma ascent through the rifting upper crust to analyze the era of magmatic activity and reveal the nature of the basement rocks in this region, as well as the record of supercontinent amalgamation events.

## Results

Zircons were separated from the three submarine volcanic rock samples collected at the three stations (C1, T9’ and R10-H2; Figs. S1 and S2). These samples are calc-alkalic rhyolites and dacite (Fig. S3) and have the most isotopically enriched compositions of all of the volcanic rocks in the OT (Fig. S4). All the U-Th-Pb–O-Hf isotope and trace element composition data for the Quaternary zircons and old zircon cores are presented in the Supplementary information.

### U–Th–Pb ages

**Figure 1:**
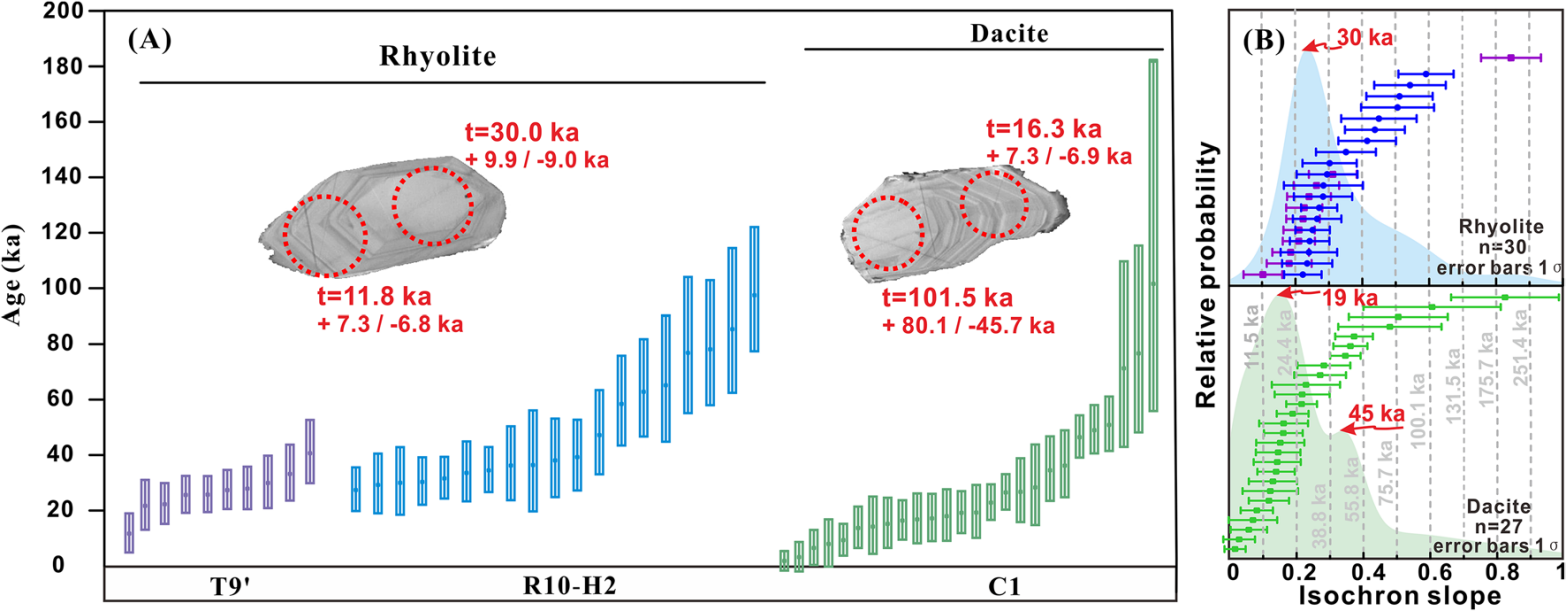
Zircon U–Th geochronology of the back-arc volcanic rocks. (**a**) CL images of two Light-CL zircons showing several microprobe analysis locations and their corresponding ages. (**b**) Cumulative PDF curves of the slopes of the ^238^U–^230^Th model isochrons for the SIMS zircon data. The purple, blue, green and vertical dashed lines represent rhyolites T9’ and R10-H2, dacite C1, and the reference ages, respectively.

**Figure 2:**
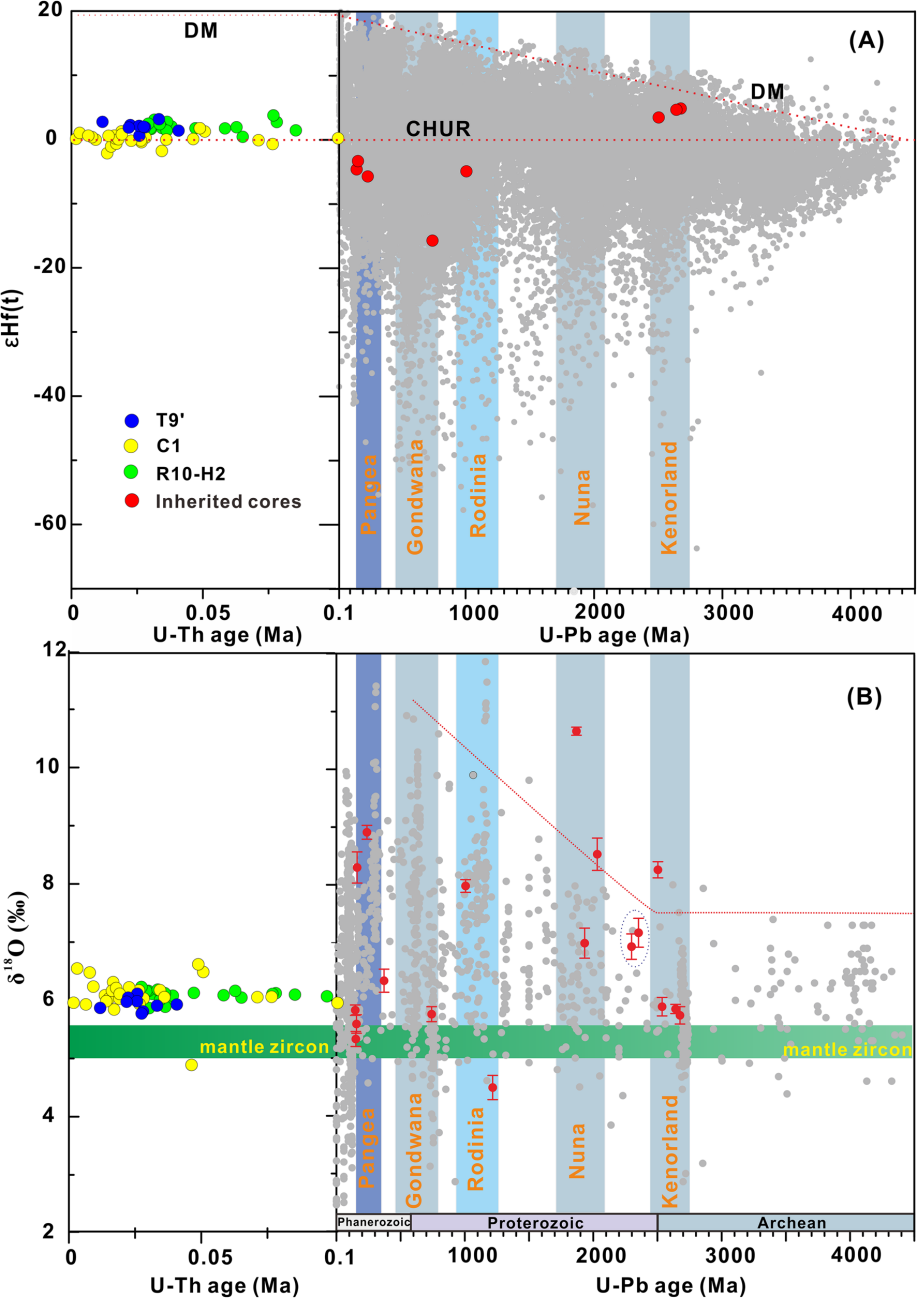
Plots of (**a**) εHf_(t)_ and (**b**) δ^18^O versus time for the Light-CL zircons and their inherited cores. The light grey dots represent global zircon εHf_(t)_ (Roberts and Spencer^43^,) and δ^18^O data (Spencer et al.^44^). The vertical bars represent supercontinent amalgamation events^35,36,44^. The dotted ellipse denotes line is mark of the inherited zircons with ages outside of the ranges of the supercontinent amalgamation events.

The CL images of the zircons can be categorized into two groups. One group is characterized by a light-CL domain in each grain (Fig. 1), and the other group has dark-CL domains surrounded by light-CL domains, i.e., inherited cores (Fig. S5). The light-CL domains are in U-Th radioactive disequilibrium (Fig. S6), and the U–Th ages of the light-CL domains from the rhyolites and the dacite are similar, with a range of ~ 100 ka (Fig. 1A; Table S1). The cumulative probability density function (PDF) curve peaks at 30 ka for the light-CL domains in the rhyolites, whereas the PDF curve peaks at 19 and 45 ka for the light-CL domains in the dacite (Fig. 1B). In contrast to the light-CL domains, the dark-CL domains in both the dacite and the rhyolites are older (U–Pb ages of 108 Ma to 2.7 Ga) (Figs. 2 and S5; Table S2), and some of the ages obtained from the dark-CL domains (Table S2) correspond to the ages of the five supercontinent amalgamation events (Fig. 2).

### Hf–O isotopes

In contrast to the dark-CL domains (Fig. 2; Table S2), the light-CL domains have narrow δ^18^O and εHf_(t)_ ranges (Figs. 2 and S7; Table S1). Seven relatively large dark-CL domains have εHf_(0)_ values of − 54.5 to − 6.8, which correspond to εHf_(t)_ values of − 15.7 to + 4.9 and two-stage crustal Hf model ages (T_DM2_) of 1419–2837 Ma (Figs. 2 and S5; Table S2). In contrast to the Proterozoic and Phanerozoic zircons, the εHf_(t)_ values of the ~ 2.7 Ga Archaean dark-CL domains are positive (Fig. 2; Table S2).

### Trace elements

**Figure 3:**
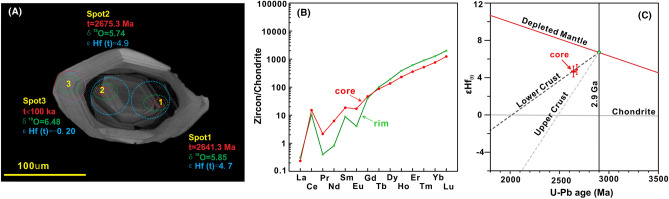
A representative zircon with an inherited core. (**a**) CL image of a zircon rim and core with the U–Th and U–Pb ages, δ^18^O and εHf_(t)_ analysis locations and data shown. (**b**) REE diagram for the zircon’s rim and core. (**c**) Plot of εHf_(t)_ versus U–Pb age for the inherited cores. The εHf_(t)_ values and ages of the geochemical reservoirs after Griffin et al.^27^, Blichert-Toft and Albarede^28^, Amelin et al.^29^. The ages and Hf–O isotopes of Spots 1 and 2 are consistent with C1@07 and C1@08 in Table S2, respectively. The age of Spot 3 is inferred from its Light-CL image, and the Hf–O isotopes of Spot 3 are consistent with TVG-C1@20 in Table S1.

The light-CL domains have high Th/U ratios (0.4–1.0; Fig. S8; Table S3). All the light-CL domains have steep chondrite-normalized rare earth element (REE) patterns with variable heavy REE (HREE) enrichments, prominent positive Ce anomalies, and strong negative Eu anomalies (Fig. S9). The dark-CL domains are less HREE enriched and do not have significant Eu anomalies (Fig. 3; Table S3).

## Discussion

### Long-lived silicic magma reservoirs beneath the OT

The light-CL domains are euhedral with oscillatory zoning (Fig. 1A), which is a robust evidence of a magmatic origin^16^. In addition, they have high Th/U ratios (> 0.4; Fig. S8), exhibiting steep chondrite-normalized REE patterns with variable HREE enrichments, prominent positive Ce anomalies, and strong negative Eu anomalies (Fig. S9), which are characteristic of magmatic zircons^16^, indicating a magmatic origin rather than a metamorphic origin^16,17^. Moreover, the light-CL domains exhibit homogeneous δ^18^O and εHf_(t)_ values (Figs. 2 and S7), suggesting that all of the light-CL domains crystallized from their parent magma^18^. Multiple age spots on individual light-CL domains with continuous and uninterrupted oscillatory zoning revealed age differences of up to 85 ka between the core and rim domains (Fig. 1A), providing further evidence of protracted zircon crystallization. Overall, the light-CL domains crystallized in a long-lived upper crustal magma reservoir for ~ 100 ka, which is consistent with the results of other studies conducted on the longevity of silicic magma systems in continental arc settings^8^. The outermost crystal rims represent the youngest phase of zircon crystallization (1.8 + 3.6/ − 3.5 ka) (Fig. 1; Table S1). We interpret this to be the approximate eruption age. Chen et al.^19^ and Huang et al.^20^ obtained U-series ages of 88.7–12.7 ka for the silicic rocks from the middle and northern OT. These ages suggest that volcanic activity has occurred throughout the OT and promoted the back-arc extension in the OT since the Late Pleistocene^19,20^. This volcanic activity was likely sustained by long-lived silicic magma reservoirs beneath the submarine volcanoes^8^.

### Origin of the zircon xenocryst cores in the silicic magmas

A prominent feature observed in this study is that some of the light-CL domains in the volcanic rocks had dark-CL domains with U–Pb ages of ~ 108 Ma to 2.7 Ga (Fig. S5; Table S2). Based on the Hf–O isotope compositions of the light-CL domains, the Quaternary volcanic rocks were likely produced by the mixing of a mantle-derived mafic magma with ~ 10–20% crust-derived silicic magma (Figs. S7C–D), overall framework of the adjacent riched radiogenic Sr–Nd isotope compositions of the whole-rock samples^6^ (Fig. S4). The involvement of a high percentage of a crustal component is consistent with the presence of old inherited zircons (the dark-CL domains) with rim overgrowths (the light-CL domains)^13^ (Fig. S5). The presence of the old inherited zircons in the subduction-related volcanic rocks is either due to the direct incorporation of the old inherited zircons into the mantle source via subducted sediments^21,22^ or the capture of fragments of old continental crust (containing the old zircons) during magma ascent^12,14,15^. Experimental results have demonstrated that the retention times of O isotopes in 20–120 μm zircons at a temperature of 900 °C are between 160 and 5700 years^23^. For hotter mantle magmas, the time required for the δ^18^O to reach diffusive equilibrium with the homogeneous mantle value is much shorter than 5.7 ka^24^. The Zr-undersaturation level of mafic melts causes the fast dissolution of pre-existing zircons under normal conditions^25^. Thus, it would not be possible for continent-derived zircon crystals that have been recycled back into the mantle by subduction to preserve highly variable O isotope signatures at mantle temperatures^24^. Therefore, the dark-CL domains with highly variable δ^18^O values (Fig. 2) must be remnants of a deeply buried basement that underwent extensive partial melting and mixing with the ascending magma. In other words, these dark-CL domains are unmelted remnants of the basement.

### Remnants of the East Asian continental blocks underlying the embryonic crustal rifting zone

During this study, we found that some of the zricons have old dark-CL inherited cores surrounded by light-CL domains (Figs. 3 and S5). As O is the relatively fast diffusing element in zircon^23^, the distinct O isotopes of the old inherited cores and the young rims (Figs. 2 and S5) suggest that the O isotopes were not reset. Therefore, not only the O isotopes, but also the Hf isotopes and REEs can reflect their original values.

Archean zircon cores were found in both the dacite and the rhyolites. The inherited Archaean zircon cores have the following characteristics. (1) They exhibit clear, bright, and broad oscillatory zoning in the CL images and euhedral to subhedral rather than oval shapes (Fig. 3A), indicating that the zircons in the igneous rocks were directly captured by the dacitic/rhyolitic magmas. (2) They have a high Th/U ratio of 0.59 (Table S3), suggesting a magmatic rather than a metamorphic origin (Th/U < 0.1)^16^. (3) They lack significant Eu anomalies, exhibit relatively flat HREE patterns (Fig. 3B), and have lower contents of incompatible elements, such as Y, Hf, and U, than the younger zircon rims (Table S3), suggesting that they crystallized from a less evolved magma^17^. (4) They have oxygen isotope compositions (5.74–5.85‰; Fig. 2B; Table S2) similar to those of mantle zircons (5.3 ± 0.3‰)^26^ and positive εHf_(t)_ values (4.7 to 4.9; Table S2) that plot between the evolutionary trends of the depleted mantle^27^ and chondrites^28^ (Fig. 3C), suggesting that the parent magma of the inherited Archaean zircon cores were mainly derived from the depleted mantle. (5) They have εHf_(t)_ values within the lower crustal range^29^ (Fig. 3C). Collectively, these lines of evidence suggest that the inherited Archaean zircon cores were derived from a mafic source that was extracted from the basaltic lower crust of the SPOT, which formed through melting of the depleted mantle ~ 2.9 Ga (Fig. 3C).

**Figure 4:**
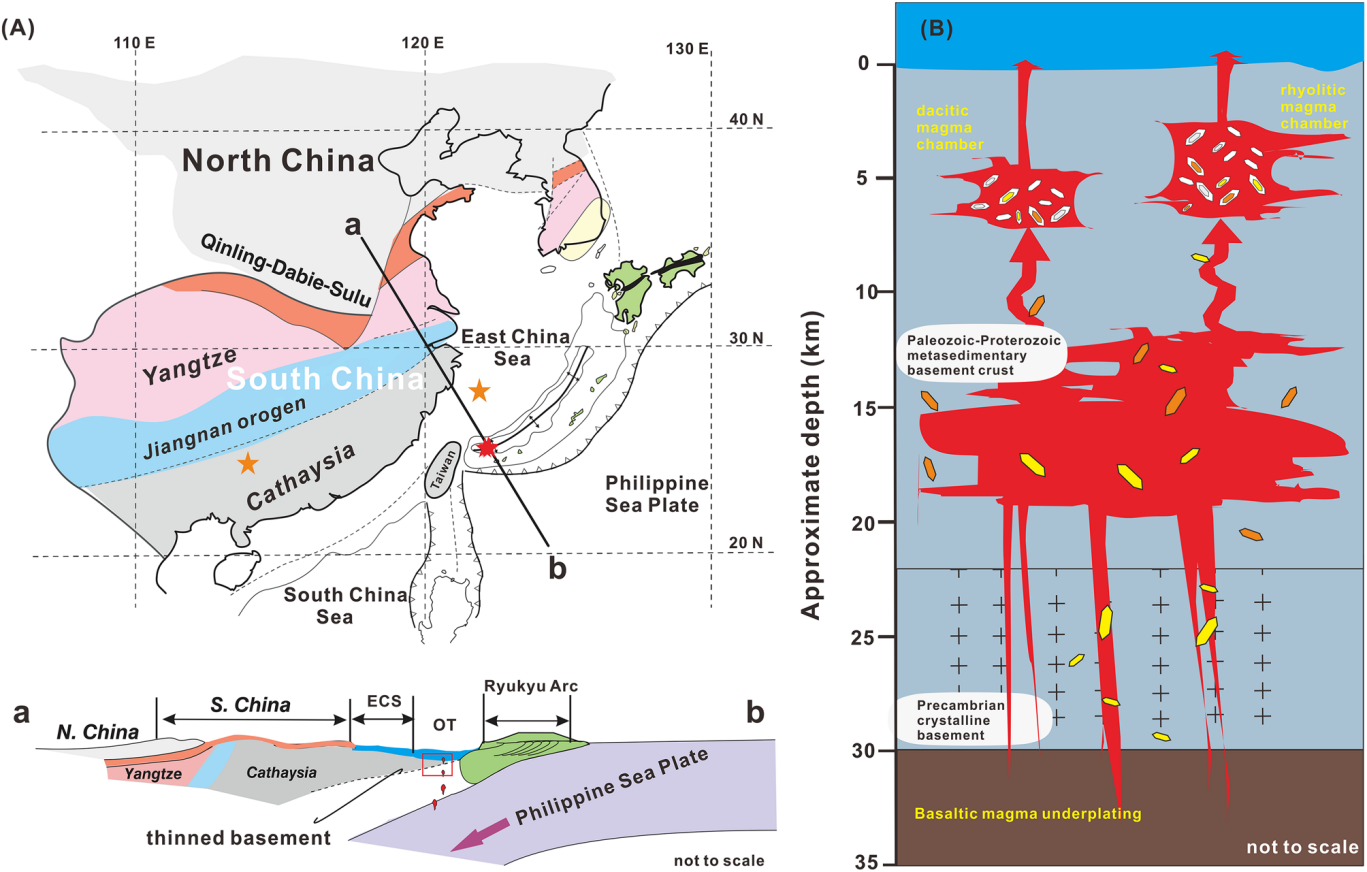
(**a**) The overall framework of the adjacent, major continental blocks and schematic profile across the OT and the China Block; modified from Isozaki^45^. The five-pointed stars show the sampling locations for this study, Li et al.^31^ and Yuan et al.^30^. (**b**) The mantle-derived magma suffered crust assimilation, and xenocrystic zircons in the crust were carried by ascending magma to shallow magma chambers. The figure was generated using the coreldraw x7 software) https://www.coreldraw.com/en/pages/coreldraw-x7/).

In addition, drilling results show that the 174 Ma granitoids in the adjacent East China Sea basin (Fig. 4A) have crustal Hf model ages of 2.9–2.5 Ga, implying that their parent magmas were derived from reworking of the Archaean lower crust^30^. Similarly, the Archaean inherited zircons (2.7–2.5 Ga) in the studied volcanic rocks suggest the presence of unexposed Archaean lower crust beneath the SPOT (Fig. 4B). The ages and Hf isotope compositions of these zircons are also similar to the zircons found in the lower crustal xenoliths in the East Asian continental blocks, southern China (Fig. 4A)^31^. Moreover, one inherited zircon with a Neoproterozoic age (741.7 Ma) has a very low εHf_(t)_ value (− 15.7) and an Hf model age (T_DM2_) of 2.6 Ga (Table S2), indicating that its parent magma was derived from the reworking of Archaean crust. Inherited zircons with East Asian continental block affinities are also abundant in the young igneous rocks of the adjacent Luzon arc^12^ and Japanese Islands^32^. Thus, the inherited Archean zircons in the volcanic rocks strongly suggest that remnants of the old East Asian continental blocks underlie the embryonic crustal rifting zone in the SPOT. In contrast to the Archaean samples, one Neoproterozoic inherited zircon (1.0 Ga) and three Mesozoic inherited zircons (150.9–237.5 Ma) exhibit slightly negative εHf_(t)_ values (− 3.3 to − 5.7) and Proterozoic Hf model ages (T_DM2_ = 1.4–2.2 Ga; Table S2), suggesting that their parent magmas were derived from the reworking of Proterozoic crustal components or from mixing of crust-derived magmas and juvenile material^27^. Thus, the older basement has experienced reworking and modification related to the addition of juvenile material since the Neoarchean. In addition, their highly variable δ^18^O values suggest that they may have originated from the metasedimentary basement in the upper and middle crust overlying the Archean basement of the lower crust (Fig. 4B).

### Inherited zircons record supercontinent amalgamation events

Notably, the age distribution of the inherited zircons coincides with five supercontinent amalgamation events (Fig. 2). We propose that the Archaean inherited zircons (2.5–2.7 Ga), with positive εHf_(t)_ values and low oxygen isotope values (5.74–5.85‰) (Fig. 2; Table S2), are unique and related to the formation of juvenile continental crust, i.e., crust that segregated rapidly from the mantle without significant involvement of older crustal materials^33^. This event occurred during the amalgamation of Kenorland and was the largest global event that affected volcanism on all continents^34^. The ages of most of the post-Archaean inherited zircons fall within the periods of supercontinent assembly at ~ 2.1–1.7 Ga (Nuna), 1.3–0.95 Ga (Rodinia), 0.7–0.5 Ga (Gondwana), and 0.35–0.18 Ga (Pangea) (Fig. 2)^35–37^. The negative εHf_(t)_ values of these inherited zircons indicate that their parent magmas contained recycled older crust^27^. Moreover, the variations in the negative εHf_(t)_ and heterogeneous high oxygen isotope values (Fig. 2) indicate a reworked older crustal source with no significant contribution from mantle-derived magma, or alternatively, they indicate different degrees of mixing between juvenile and evolved sources^38^. The latter interpretation may be consistent with the fact that during supercontinent assembly, the continental crust with nonradiogenic Hf isotopes was subducted into the mantle, which resulted in more crust-mantle interaction and led to the negative Hf isotopes of the parent magma of the zircons^38^. As such, the inherited zircons from the OT act as a record of global continent amalgamation events, which have also been found in other arc-related volcanic rocks^12,14,15^.

## Conclusions

The zircon U–Th–Pb ages, trace element compositions, and Hf–O isotopes support the following model for the two silicic magma eruptions in the southwest OT. (1) There were long-lived (at least 100 ka) magma reservoirs beneath the submarine volcanos, indicating that the time of OT rifting is at least 100 ka . (2) The zircon xenocrysts were acquired during the ascent of mantle-derived magma through the overlying crust rather than from recycled subducted sediments. (3) The occurrence of these Archean zircons suggests the presence of unexposed Archean materials in the crust of the southern OT, which have experienced complex modifications related to the addition of juvenile material and the reworking from the Neoarchean onward. (4) The inherited zircons record five supercontinent amalgamation events.

## Methods

The zircons were separated from the three volcanic rock samples (Fig. S2). Representative zircon grains were mounted in epoxy resin and were polished to expose the grain’s centers. For all the epoxy mounts of the zircon grains, cathodoluminescence (CL) images were obtained prior to analysis and were used to guide the analysis locations. First, secondary ion mass spectrometry (SIMS) analysis was performed on the zircons to obtain U–Th–Pb–O isotopic and trace element data. The Lu–Hf isotopes of the same zircon grains were then analysed via LA-MC-ICPMS.

The zircons ^238^U–^230^Th disequilibrium ages and trace element compositions were obtained using the CAMECA IMS 1270 SIMS at the University of California Los Angeles (UCLA) following the analytical protocols described by Schmitt et al.^39^ and Bell and Harrison^40^. Zircon U–Pb and O analyses were performed using the CAMECA IMS 1280 at the Institute of Geology and Geophysics, Chinese Academy of Sciences (IGGCAS) following the analytical procedures described by Li et al.^41^ and Tang et al.^42^. Finally, Hf isotope analysis was carried out on the zircons using the LA-MC-ICP-MS at Nanjing University, China. The details of the analytical methods and zircon U–Th–Pb–O–Hf isotopic and trace element data are presented in the Supplementary Information.

## Supplementary information

Supplementary Information.

## Data Availability

All the data are reported in the Supplementary Information.
