# Supplementary material for: Zircon record of an Archaean crustal fragment and supercontinent amalgamation in quaternary back-arc volcanic rocks
Source: Sci Rep. 2021 Jun 11;11:12367. doi: 10.1038/s41598-021-90578-9 (PMC8196102; doi:10.1038/s41598-021-90578-9)
Supplement: Supplementary file 1 — Supplementary Information. [file 41598_2021_90578_MOESM1_ESM.docx]

**Supplementary information for**

Zircon record of an Archaean crustal fragment and supercontinent amalgamation in Quaternary back-arc volcanic rocks

Zhi-gang Zeng^1, 2, 3,4 *^, Zu-xing Chen^1,*^, Yu-xiang Zhang^1^

1 Key Laboratory of Marine Geology and Environment, Institute of Oceanology, Chinese Academy of Sciences, Qingdao 266071, China,

2 Laboratory for Marine Mineral Resources, Qingdao National Laboratory for Marine Science and Technology, Qingdao 266071, China,

3 Center for Ocean Mega-Science, Chinese Academy of Sciences, Qingdao 266071, China,

4 University of Chinese Academy of Sciences, Beijing 100049, China

Corresponding author: Z.-G. Zeng (zgzeng@ms.qdio.ac.cn); Z.-X. Chen (chenzuxing@qdio.ac.cn)

Contents of this file

This supplementary materials file contains extended descriptions of the analytical methods, Figures S1 to S9 and Tables S1 to S8.

Supplementary analytical methods

***Zircon separation and preparation***

The volcanic rock samples that are described in this paper were recovered using the television grab method during a cruise of the R/V KEXUE HAO in 2014 and were collected at stations T9' (rhyolite), C1 (dacite), and R10-H2 (rhyolite) (Fig. S1) in the SPOT. The C1 dacite is Si-unsaturated and the T9' and R10-H2 rhyolites lack rutile as was reported by Chen et al.^1, 2^. The monazite at T9', C1, and R10-H2 is not saturated as was reported by Chen et al.^1, 2^. Before separating the zircons, the volcanic rock samples (Fig. S2) were washed to remove any adhering sediments. Then, they were cut into smaller pieces (1×1 cm), and any weathered rinds and adhering sediments were removed. The fresh fragments were cleaned in an ultrasonic bath for approximately 3 hours. The water in the bath was changed every 20 to 30 minutes. After these steps, the possible contamination of the detrital zircons in the adhering sediments was eliminated. Finally, after drying in an oven at 60°C for 24 hours, all the grains were pulverized to 200 mesh in an agate mill before separating out the zircons.

The zircons were separated using standard density and magnetic techniques, and then they were manually selected under a binocular microscope. Representative zircon grains were mounted in epoxy resin and polished to expose their grain centres. To guide the selection of the in situ analysis spots, cathodoluminescence (CL) images were obtained to reveal the external morphologies and internal structures of the zircons. The CL images were obtained using a HITACHI S3000-N scanning electron microscope at Nanjing Hong-Chuang Geological Exploration Technology Service Company.

***Zircon ^238^U-^230^Th dating***

^238^U–^230^Th disequilibrium dating was performed using the CAMECA IMS 1270 SIMS at UCLA following the analytical procedures described by Schmitt et al.^3^. All the zircon crystals were mounted in epoxy and polished to reveal flat surfaces, which were coated with a conductive 20 to 30 nm layer of Au prior to analysis. The large zircon grains (mostly length > 100 μm) in the samples were chosen for in situ analysis (using a 25 to 30 μm spot size). The ion microprobe ^230^Th/^232^Th and ^238^U/^232^Th analyses conducted using the CAMECA IMS 1270 routinely achieved a relative precision and accuracy of approximately 1–2 %, with a spot size of approximately 50 μm and a depth resolution of <3 μm in the single electron multiplier collector peak-jumping mode. The accuracy was monitored by interspersed analyses of the AS3 and Qinghu zircon standards (Liu et al.^4^). The former yielded a unity secular equilibrium ratio of ^230^Th/^238^U = 1.022 ±0.016 (activities denoted in parentheses; mean squared weighted deviation (MSWD) = 0.5; n = 6; Table S4), and the latter yielded a unity secular equilibrium ratio of ^230^Th/^238^U = 1.098 ± 0.015 (MSWD = 2.2; n = 25; Table S4). The crystallization age was calculated using a correction scheme in Python code using the measured zircon ^238^U/^232^Th ratio and the U/Th partition coefficient ratio between zircon and melt^5^.

***Zircon trace element analysis***

The zircon trace element abundances were acquired using the CAMECA IMS 1270 ion probe (SIMS) at UCLA. Secondary ions were generated by bombarding the sample surface with a mass-filtered ^16^O^-^ primary ion beam at a current of approximately 60 nA focused on a 25×30 μm oval spot. The secondary ion accelerating voltage was 10 kV, and the secondary ions with an energy bandpass of 50 eV were analysed at a mass resolution (m/△m) of approximately 4500. The combined secondary magnet settling and dwell time per mass cycle was approximately 45 s, and 100 cycles were acquired per individual profile. These conditions resulted in depth intervals of approximately 0.2 μm per cycle, with a total depth of approximately 20 μm for an individual profile. The sensitivity factors were calibrated using the analysis of the National Institute of Standard and Technology (NIST) standard glasses and the 91500 zircon standard. Further details about the zircon trace element analysis are described by Bell and Harrison^6^.

***Zircon U-Pb dating***

The U-Pb isotope compositions were analysed using the CAMECA IMS 1280HR SIMS at the IGGCAS, Beijing. The instrument description and analytical procedures can be found in Li et al.^7^, and only a brief summary is given here. The primary O_2_^–^ ion beam spot was approximately 15×10 μm in size. The positive secondary ions were extracted with a 10 kV potential. In the secondary ion beam optics, a 60 eV energy window was used, together with a mass resolution of approximately 5400 (at a 10% peak height), to separate the Pb^+^ peaks from the isobaric interferences. A single electron multiplier was used in the ion-counting mode to measure the secondary ion beam’s intensity in peak-jumping mode. Analyses of zircon standard Plesovice were interspersed with the analyses of the unknown grains. Each measurement consisted of 7 cycles. Pb/U calibration was performed relative to the zircon standard Plesovice (^206^Pb/^238^U age = 337 Ma; Sláma et al.^8^). The U and Th concentrations were calibrated using zircon standard 91500 (Th = 29 ppm, and U = 81 ppm; Wiedenbeck et al.^9^). A long-term uncertainty of 1.5% (1σ relative standard deviation (RSD)) for the ^206^Pb/^238^U measurements of the standard zircons was propagated to the unknowns (Li et al.^10^), although the measured ^206^Pb/^238^U error in a specific session was generally ≤1% (1σ RSD). The measured compositions were corrected for common Pb using nonradiogenic ^204^Pb. The corrections were quite small and were insensitive to the choice of the common Pb composition. Thus, the average of the present-day crustal composition (Stacey and Kramers^11^) was used for the common Pb, assuming that this Pb was largely surface contamination acquired during sample preparation. The data reduction was carried out using the Isoplot/Ex v. 2.49 program (Ludwig^12^). The concordia U-Pb ages are quoted with 95% confidence intervals, except where noted otherwise. To monitor the external uncertainties in the SIMS U-Pb zircon dating calibrated using the Plesovice standard, an in-house zircon standard (Qinghu) was also analysed as an unknown along with the other unknown zircons. Thirty-four measurements of the Qinghu zircon (Table S5) yielded a concordia age of 159.1 ±1.5 Ma, which is identical within the error margin to the recommended value of 159.5 ± 0.2 Ma (Li et al.^13^).

***Zircon O analysis***

The in situ zircon oxygen isotope compositions were analysed on the same grains that were previously dated using the CAMECA IMS 1280 at the IGGCAS. The details of the analytical procedures are described by Tang et al.^14^. The mount was repolished to remove any oxygen implanted in the zircon surface during the U-Pb dating process. The Cs^+^ primary ion beam was accelerated at 10 kV at an intensity of approximately 2 nA. The spot size was approximately 20 μm in diameter. The normal-incidence electron flood gun was used to compensate for sample charging during analysis with a homogeneous electron density across a 100- μm oval area. Nuclear magnetic resonance (NMR) was applied to stabilize the magnetic field. The oxygen isotope compositions were measured using the multi-collection mode on two off-axis Faraday cups. The measured oxygen isotope ratios are reported in standard per mil notation relative to Vienna standard mean ocean water (V_SMOW_, ^18^O/^16^O = 0.0020052; Faghihi et al.^15^). The instrumental mass fraction (IMF) factor was corrected through replicate analysis of zircon standard Penglai with a δ^18^O value of 5.3‰^16^. The internal precision of a single analysis was generally greater than 0.20‰ (2σ standard error) for the ^18^O/^16^O ratio. During the course of this study, the in-house zircon standard Qinghu was also analysed as an unknown along with the other unknowns. The measurements of the Qinghu zircon yielded a weighted mean value that is consistent with the reported value of 5.4 ± 0.2‰ within the error margin (Li et al.^13^) (Table S6). All of the in situ oxygen isotope analysis data are presented in Tables S1 and S2.

***Zircon Lu–Hf isotope measurements***

After the SIMS zircon U-Pb dating and O isotope and trace element analyses, the zircon in situ Hf isotope analysis was carried out using the LA-MC-ICP-MS at Nanjing University, Nanjing, China. The measurements were performed on polished, sectioned zircon grains mounted in epoxy resin, and the analysis locations were guided by the CL and transmitted light images and U-Pb age data. The measurements involved ablating the shallow pits generated by the preceding SIMS analysis. These analyses were conducted with a beam diameter of 44 μm, a repetition rate of 8 Hz, and a laser power of approximately 15 J/cm^2^. The aerosols ablated by the laser were transported to the mass spectrometer for isotopic determination using He as the carrier gas. All of the Lu-Hf isotope results are reported with an error of 2σ of the mean. Zircons 91500 and Mud Tank were used as the reference standards during our routine analyses, yielding ^176^Hf/^177^Hf ratios of 0.282291±0.000014 (2SD, n=31; Table S7) and 0.282488±0.000009 (2SD, n=31; Table S7), respectively. These values are consistent with the recommended ^176^Hf/^177^Hf ratios of 0.282306±0.000010 for zircon 91500 (Woodhead et al.^17^) and 0.282507 ±0.000006 for the Mud Tank zircon (Woodhead and Hergt^18^) within the analytical error margins.

Supplementary Figures

**
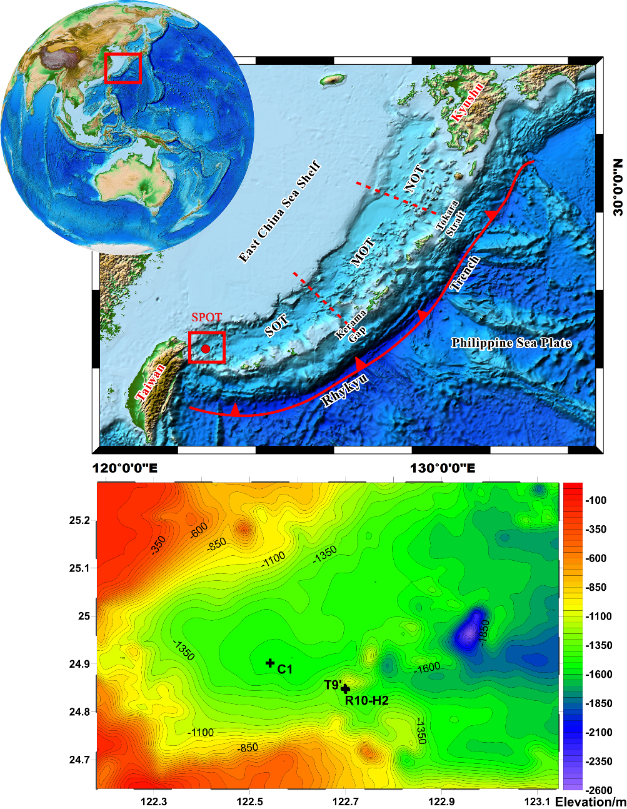
**

**Figure S1.** Geologic map of the Okinawa Trough (OT), which is comprised of three segments: Northern OT (NOT); Middle OT (MOT); Southern OT (SOT). The global inset in the upper left corner shows the location of the OT in the western Pacific. The [red](javascript:;) [box](javascript:;) in the southernmost part of the OT (SPOT) is the study area. The details of the study area and the sampling locations are shown on the bathymetric map.

**
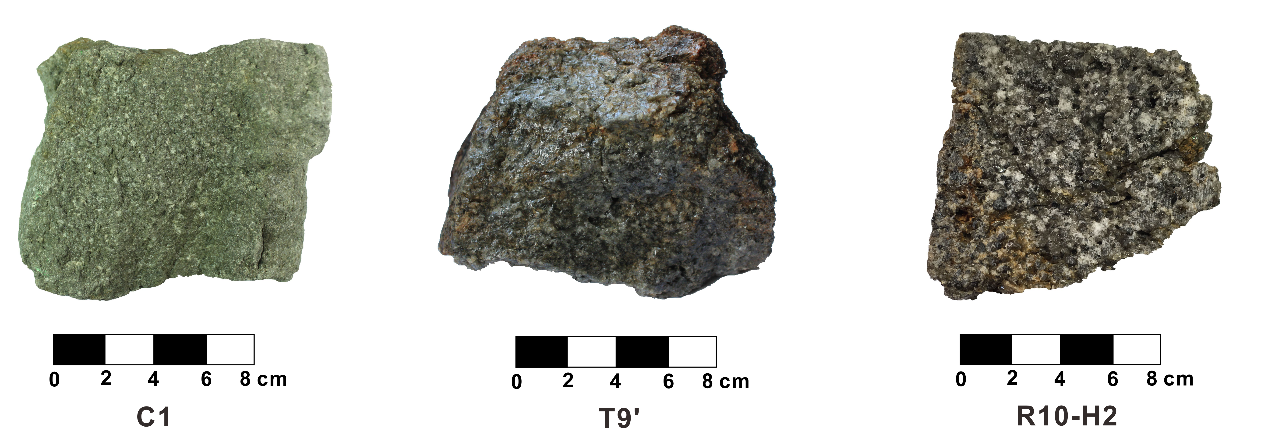
**

**Figure S2.** Hand specimen photographs of the studied samples.

**
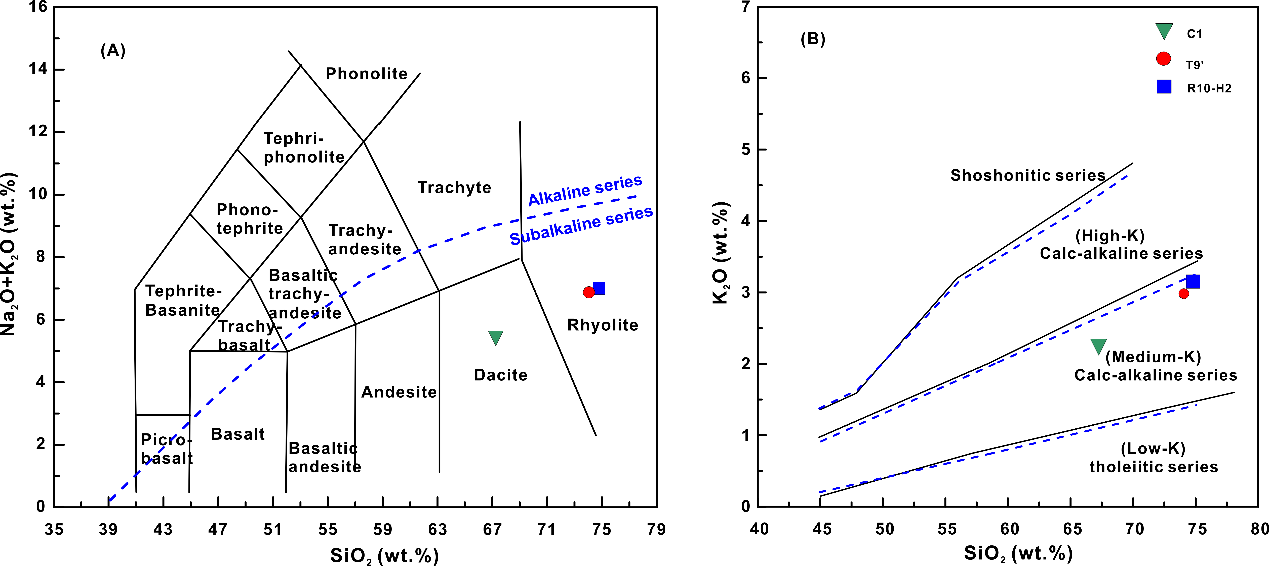
**

**Figure S3.** Classification diagrams for the studied rocks from the SPOT. (A) Total alkalis versus silica diagram [(Na_2_O+K_2_O) vs. SiO_2_)] of Bas et al.^19^. (B) K_2_O versus SiO_2_; the boundaries are after Roberts and Clemens^20^.


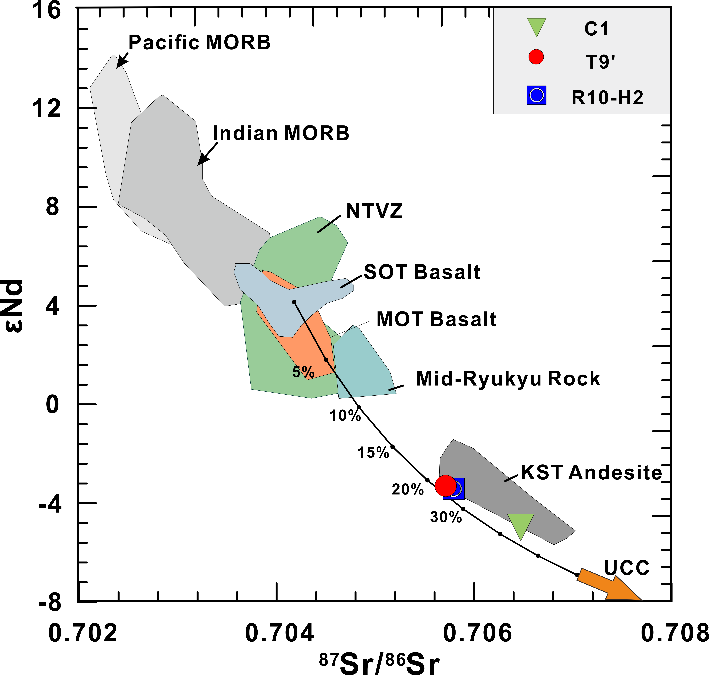


**Figure S4.** ^87^Sr/^86^Sr versus ^143^Nd/^144^Nd diagram for the studied volcanic rocks; the fields are after Chen et al.^2^. NTVZ = northern Taiwan Volcanic Zone. KST= Kueishantao islet. MORB= mid-ocean ridge basalt. UCC= upper continental crust.


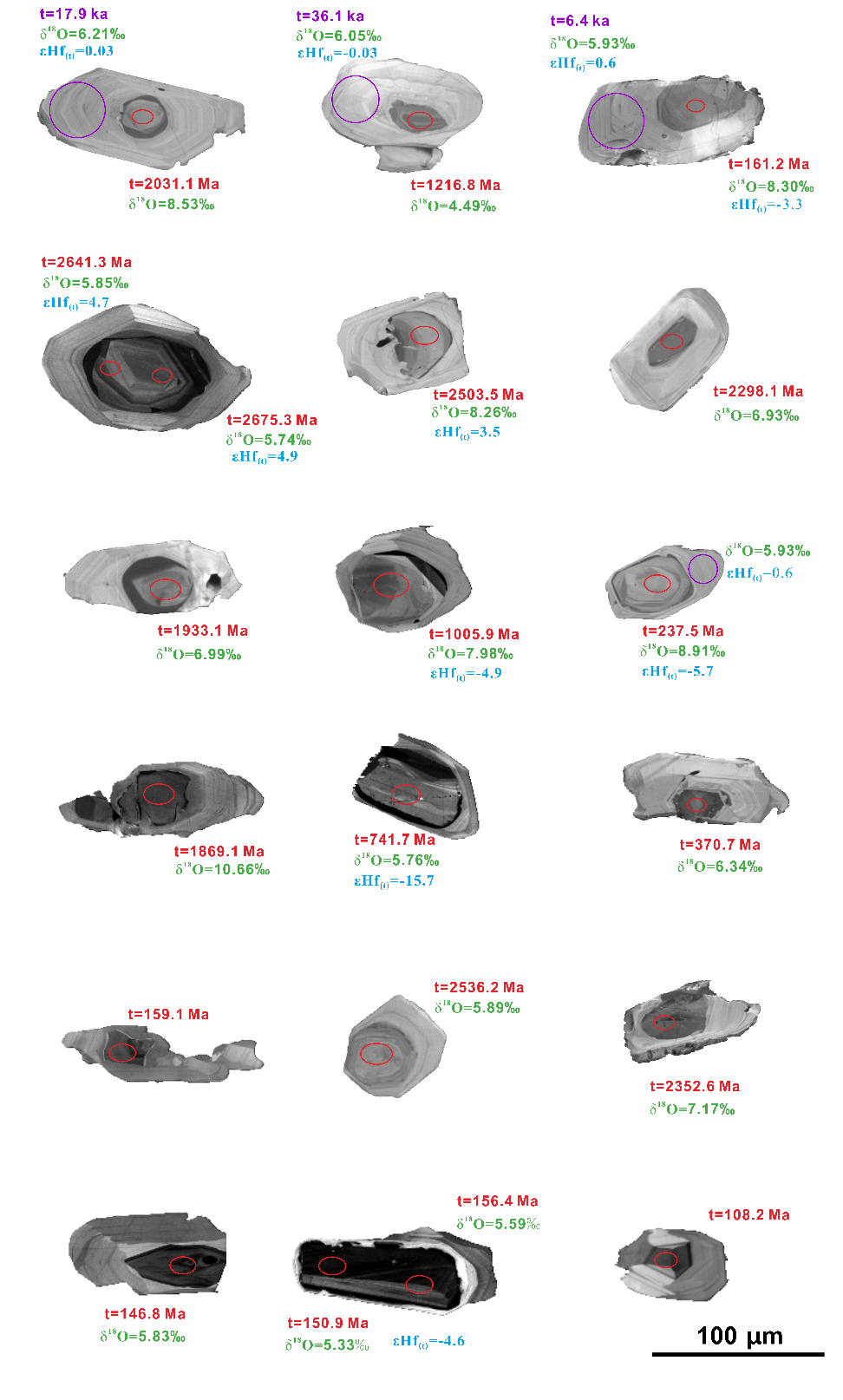


**Figure S5.** CL images of dark-CL zircon cores enveloped in light-CL domains with SIMS ages, δ^18^O isotope compositions, and Hf isotope data.

**
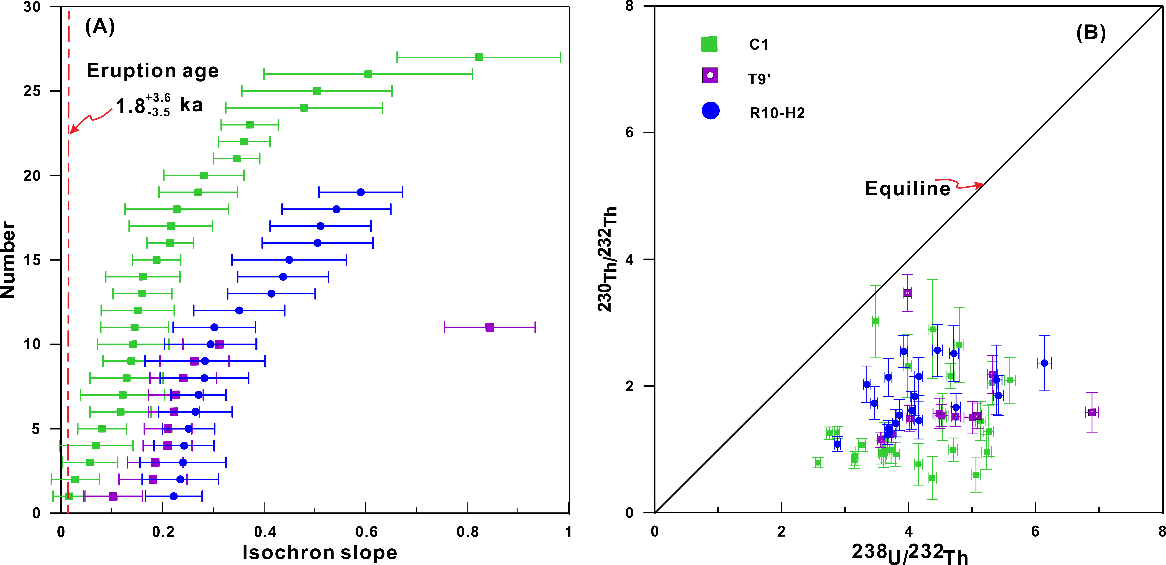
**

**Figure S6.** (A) Rank order plots of the slopes of the ^238^U–^230^Th model (slope + 1 s output by the code of Boehnke et al.^5^ showing an extensive range from near the volcanic eruption age to near the equiline. (B) ^230^Th/^232^Th versus ^238^U/^232^Th activity ratio diagram for the SIMS Light-CL zircon domain analyses of the dacite and rhyolite samples.


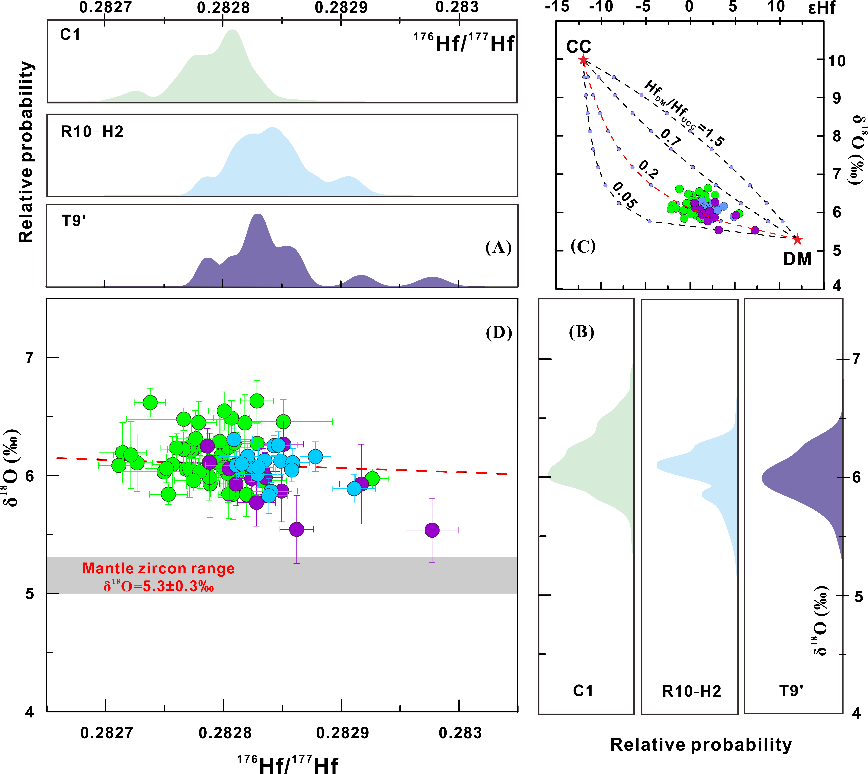


**Figure S7.** Oxygen and hafnium isotope systematics of the Light-CL zircon domains. A−B: The oxygen and hafnium isotopic heterogeneities in the Light-CL zircon domains are displayed as kernel density estimates. C−D: Oxygen-hafnium isotope covariance-based models of the mixing between mantle and crustal sources. The end member compositions of the zircons from the depleted mantle (DM; εHf = 12, δ^18^O = 5.6‰) and the continental crust (CC; εHf = −12, δ^18^O = 10‰) are from Li et al.^21^.


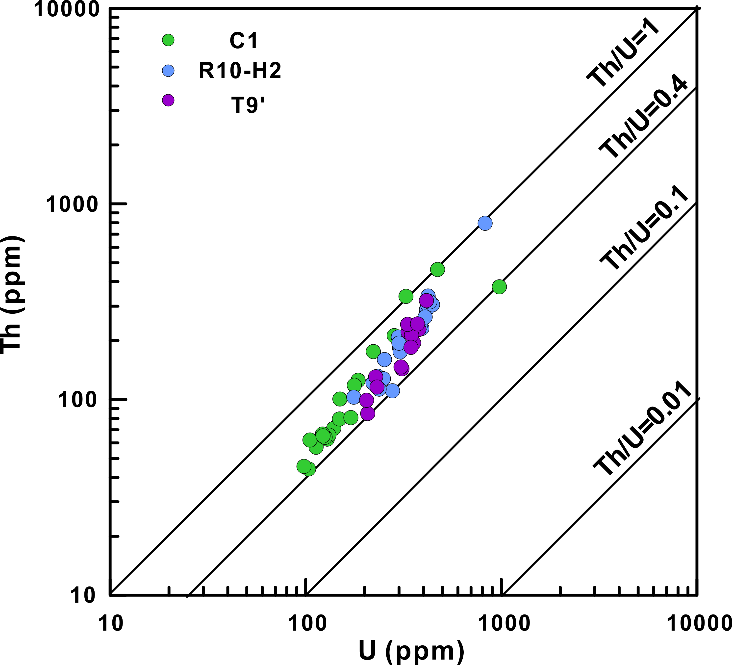


**Figure S8.** Th-U plot for the Light-CL zircon domains from the studied samples. The Light-CL zircon domains in the back-arc volcanic rock samples have high Th/U ratios (0.4−1.0).


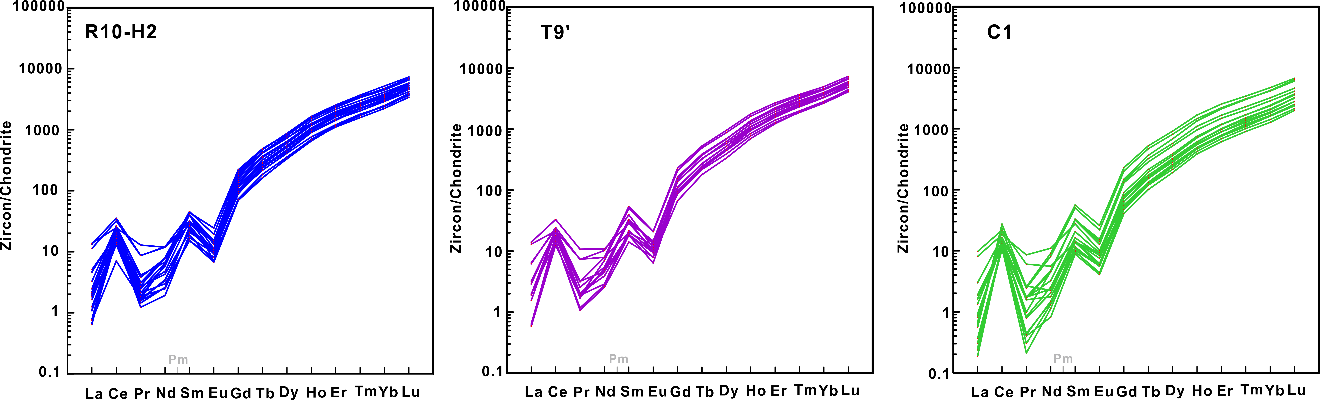


**Figure S9.** Rare earth element (REE) concentrations of the Light-CL zircon domains normalized to chondrite^22^.

Supplementary tables

**Table S1. SIMS zircon U-Th and O analyses and corresponding LA-MC-ICP-MS Hf isotopic analysis results for the Light-CL zircons in the volcanic rocks.**

| ***Sample*** | ***(^238^U/^232^Th)*** | ***1s*** | ***(^230^Th/^232^Th)*** | ***1s*** | ***Th age*** | ***pos 1s*** | ***neg 1s*** | ***U*** | ^176^Hf/^177^Hf | 2SE | εHf | δ**^18^O** | **2SE** |
| --- | --- | --- | --- | --- | --- | --- | --- | --- | --- | --- | --- | --- | --- |
| R10-H2-2@1.ais | 4.05 | 0.06 | 1.61 | 0.29 | 38.1 | 15.1 | -13.2 | 68 | 0.282829 | 0.000016 | 2.02 | 6.01 | 0.11 |
| R10-H2-2@2.ais | 3.68 | 0.05 | 2.14 | 0.30 | 78.2 | 24.8 | -20.2 | 113 | 0.282849 | 0.000014 | 2.71 | 6.12 | 0.12 |
| R10-H2-3@3.ais | 3.68 | 0.06 | 1.32 | 0.14 | 31.6 | 7.7 | -7.3 | 153 | 0.282859 | 0.000014 | 3.07 | 6.11 | 0.12 |
| R10-H2-4@4.ais | 4.75 | 0.07 | 1.66 | 0.22 | 30.3 | 8.9 | -8.2 | 110 | 0.282818 | 0.000015 | 1.62 | 5.87 | 0.07 |
| R10-H2-5@5.ais | 5.39 | 0.09 | 2.09 | 0.55 | 36.4 | 19.7 | -16.7 | 44 | 0.282849 | 0.000014 | 2.74 | 5.89 | 0.12 |
| R10-H2-6@6.ais | 2.88 | 0.05 | 1.08 | 0.12 | 34.5 | 8.4 | -7.8 | 270 | 0.282822 | 0.000014 | 1.77 | 6.01 | 0.11 |
| R10-H2-8@7.ais | 4.71 | 0.07 | 2.51 | 0.45 | 65.2 | 25.0 | -20.4 | 75 | 0.282784 | 0.000013 | 0.41 | 6.05 | 0.10 |
| R10-H2-10@8.ais | 3.93 | 0.06 | 2.55 | 0.25 | 97.5 | 24.6 | -20.0 | 309 | - | - | - | 6.07 | 0.17 |
| R10-H2-11@9.ais | 3.85 | 0.06 | 1.54 | 0.25 | 39.3 | 13.5 | -12.0 | 172 | - | - | - | 6.08 | 0.13 |
| R10-H2-13@11.ais | 3.46 | 0.05 | 1.73 | 0.26 | 58.4 | 17.4 | -15.0 | 276 | 0.282821 | 0.000018 | 1.72 | 6.09 | 0.12 |
| R10-H2-14@12.ais | 3.34 | 0.05 | 2.03 | 0.29 | 85.4 | 29.1 | -22.9 | 137 | 0.282812 | 0.000023 | 1.41 | 6.10 | 0.09 |
| R10-H2-15@13.ais | 4.46 | 0.08 | 2.56 | 0.41 | 76.9 | 27.2 | -21.8 | 112 | 0.282878 | 0.000016 | 3.75 | 6.11 | 0.12 |
| R10-H2-15-2@14.ais | 6.14 | 0.11 | 2.36 | 0.44 | 36.2 | 14.1 | -12.5 | 104 | 0.282810 | 0.000012 | 1.35 | 6.12 | 0.12 |
| R10-H2-15-3@15.ais | 4.10 | 0.06 | 1.84 | 0.31 | 47.2 | 16.2 | -14.1 | 114 | 0.282821 | 0.000014 | 1.74 | 6.13 | 0.09 |
| R10-H2-16@16.ais | 4.16 | 0.06 | 1.46 | 0.29 | 30.0 | 12.9 | -11.5 | 62 | 0.282821 | 0.000026 | 1.72 | 6.16 | 0.11 |
| R10-H2-17@17.ais | 5.42 | 0.08 | 1.85 | 0.32 | 29.2 | 11.3 | -10.2 | 101 | 0.282841 | 0.000012 | 2.45 | 6.16 | 0.13 |
| R10-H2-18@18.ais | 4.16 | 0.06 | 2.15 | 0.30 | 62.8 | 18.9 | -16.1 | 130 | 0.282826 | 0.000015 | 1.90 | 6.16 | 0.11 |
| R10-H2-19@19.ais | 3.80 | 0.06 | 1.41 | 0.22 | 33.6 | 11.3 | -10.3 | 161 | 0.282839 | 0.000014 | 2.39 | 6.18 | 0.56 |
| R10-H2-21@21.ais | 3.68 | 0.05 | 1.24 | 0.16 | 27.4 | 8.2 | -7.6 | 118 | 0.282807 | 0.000013 | 1.25 | 6.23 | 0.69 |
| C1-1@1.ais | 5.60 | 0.08 | 2.09 | 0.36 | 34.4 | 12.2 | -11.0 | 46 | 0.282721 | 0.000015 | -1.79 | 6.17 | 0.28 |
| C1-2@2.ais | 4.53 | 0.07 | 1.51 | 0.37 | 28.2 | 15.5 | -13.6 | 76 | 0.282780 | 0.000016 | 0.28 | 6.03 | 0.14 |
| C1-3@3.ais | 3.63 | 0.05 | 1.19 | 0.23 | 26.6 | 12.1 | -10.9 | 137 | 0.282760 | 0.000015 | -0.43 | 5.94 | 0.13 |
| C1-5@5.ais | 3.27 | 0.05 | 1.07 | 0.10 | 26.4 | 6.6 | -6.2 | 112 | 0.282767 | 0.000019 | -0.16 | 6.17 | 0.12 |
| ***TableS1 (continued)*** |  |  |  |  |  |  |  |  |  |  |  |  |  |
| ***Sample*** | ***(^238^U/^232^Th)*** | ***1s*** | ***(^230^Th/^232^Th)*** | ***1s*** | ***Th age*** | ***pos 1s*** | ***neg 1s*** | ***U*** | ^176^Hf/^177^Hf | 2SE | εHf | δ**^18^O** | **2SE** |
| C1-6@6.ais | 3.80 | 0.06 | 0.92 | 0.18 | 13.6 | 7.7 | -7.2 | 139 | 0.282711 | 0.000017 | -2.15 | 6.09 | 0.15 |
| C1-7@7.ais | 3.99 | 0.06 | 2.32 | 0.50 | 76.6 | 38.7 | -28.5 | 205 | 0.282752 | 0.000018 | -0.72 | 6.06 | 0.12 |
| C1-9@9.ais | 3.60 | 0.05 | 0.92 | 0.20 | 15.1 | 9.4 | -8.7 | 106 | 0.282741 | 0.000014 | -1.10 | 6.03 | 0.10 |
| C1-10@10.ais | 2.88 | 0.04 | 1.27 | 0.10 | 46.3 | 7.9 | -7.4 | 291 | 0.282774 | 0.000016 | 0.06 | 4.89 | 0.99 |
| C1-11@11.ais | 5.06 | 0.08 | 0.60 | 0.28 | 1.8 | 3.6 | -3.5 | 74 | 0.282775 | 0.000016 | 0.10 | 5.96 | 0.09 |
| C1-12@12.ais | 4.71 | 0.07 | 0.99 | 0.19 | 9.2 | 5.9 | -5.6 | 350 | 0.282771 | 0.000017 | -0.03 | 6.24 | 0.09 |
| C1-13@13.ais | 4.16 | 0.06 | 0.76 | 0.33 | 7.9 | 8.9 | -8.2 | 87 | 0.282782 | 0.000013 | 0.35 | 6.23 | 0.12 |
| C1-13-2@14.ais | 3.16 | 0.05 | 0.89 | 0.14 | 19.1 | 7.8 | -7.3 | 173 | 0.282807 | 0.000014 | 1.25 | 6.03 | 0.10 |
| C1-14@15.ais | 3.58 | 0.05 | 0.96 | 0.19 | 17.1 | 8.9 | -8.2 | 84 | 0.282753 | 0.000016 | -0.67 | 5.84 | 0.09 |
| C1-19@19.ais | 4.66 | 0.07 | 2.16 | 0.19 | 50.8 | 10.2 | -9.4 | 237 | 0.282807 | 0.000015 | 1.23 | 6.49 | 0.15 |
| C1-20@20.ais | 3.73 | 0.06 | 0.99 | 0.22 | 16.7 | 9.4 | -8.6 | 129 | 0.282776 | 0.000015 | 0.16 | 6.31 | 0.17 |
| C1-22@21.ais | 4.37 | 0.06 | 0.55 | 0.34 | 3.1 | 5.5 | -5.2 | 94 | 0.282801 | 0.000013 | 1.02 | 6.55 | 0.16 |
| C1-23@22.ais | 2.75 | 0.04 | 1.25 | 0.10 | 48.9 | 9.1 | -8.4 | 186 | 0.282821 | 0.000015 | 1.75 | 6.62 | 0.12 |
| [C1-25@23.ais](mailto:C1-25@23.ais) | 2.57 | 0.04 | 0.79 | 0.08 | 22.8 | 6.6 | -6.2 | 238 | 0.282766 | 0.000014 | -0.21 | 6.22 | 0.15 |
| C1-1-7@1.ais | 4.80 | 0.07 | 2.65 | 0.60 | 71.2 | 38.4 | -28.4 | 52 | 0.282769 | 0.000022 | -0.12 | 6.05 | 0.17 |
| [C1-1-10@2.ais](mailto:C1-1-10@2.ais) | 5.23 | 0.08 | 0.96 | 0.27 | 6.4 | 6.5 | -6.1 | 95 | 0.282789 | 0.000014 | 0.59 | 5.93 | 0.28 |
| [C1-1-14@3.ais](mailto:C1-1-14@3.ais) | 3.14 | 0.05 | 0.83 | 0.13 | 16.3 | 7.3 | -6.9 | 182 | 0.282789 | 0.000014 | 0.59 | 6.16 | 0.35 |
| [C1-2-8@1.ais](mailto:C1-2-8@1.ais) | 3.64 | 0.06 | 0.99 | 0.21 | 17.9 | 9.7 | -8.9 | 79 | 0.282773 | 0.000013 | 0.03 | 6.21 | 0.26 |
| C1-2-11@2.ais | 5.32 | 0.08 | 2.05 | 0.34 | 36.1 | 12.7 | -11.4 | 103 | 0.282771 | 0.000015 | -0.03 | 6.05 | 0.24 |
| C1-2-9@3.ais | 5.27 | 0.08 | 1.29 | 0.40 | 14.1 | 10.8 | -9.8 | 66 | 0.282788 | 0.000013 | 0.56 | 5.98 | 0.19 |
| C1-2-5@4.ais | 5.14 | 0.08 | 1.45 | 0.31 | 19.2 | 9.9 | -9.1 | 176 | 0.282794 | 0.000016 | 0.79 | 6.11 | 0.15 |
| C1-1-15@4.ais | 4.38 | 0.07 | 2.90 | 0.78 | 101.5 | 80.1 | -45.7 | 200 | 0.282777 | 0.000013 | 0.18 | 5.96 | 0.16 |
| ***Sample*** | ***(^238^U/^232^Th)*** | ***1s*** | ***(^230^Th/^232^Th)*** | ***1s*** | ***Th age*** | ***pos 1s*** | ***neg 1s*** | ***U*** | ^176^Hf/^177^Hf | 2SE | εHf | δ**^18^O** | **2SE** |
| T9'-2@2.ais | 4.02 | 0.06 | 1.49 | 0.21 | 33.3 | 10.5 | -9.6 | 169 | 0.282861 | 0.000017 | 3.16 | 5.91 | 0.40 |
| T9'-3@3.ais | 3.56 | 0.06 | 1.15 | 0.12 | 25.8 | 6.7 | -6.3 | 151 | 0.282788 | 0.000015 | 0.57 | 6.11 | 0.25 |
| T9'-4@4.ais | 5.32 | 0.08 | 2.18 | 0.30 | 40.8 | 12.0 | -10.8 | 190 | 0.282811 | 0.000014 | 1.37 | 5.93 | 0.18 |
| T9'-6@5.ais | 5.08 | 0.07 | 1.53 | 0.20 | 22.4 | 7.6 | -7.1 | 368 | 0.282835 | 0.000015 | 2.24 | 6.05 | 0.23 |
| T9'-7@6.ais | 4.74 | 0.07 | 1.52 | 0.16 | 25.7 | 6.9 | -6.5 | 274 | 0.282832 | 0.000014 | 2.12 | 5.98 | 0.21 |
| T9'-9@7.ais | 4.52 | 0.10 | 1.53 | 0.17 | 28.0 | 7.9 | -7.4 | 259 | 0.282825 | 0.000014 | 1.89 | 5.81 | 0.16 |
| T9'-8@8.ais | 3.74 | 0.06 | 1.24 | 0.13 | 27.5 | 7.3 | -6.8 | 307 | 0.282828 | 0.000016 | 1.98 | 5.77 | 0.20 |
| T9'-10@9.ais | 5.01 | 0.14 | 1.50 | 0.26 | 21.8 | 9.3 | -8.6 | 130 | 0.282824 | 0.000014 | 1.84 | 5.97 | 0.22 |
| T9'-11@10.ais | 6.89 | 0.10 | 1.59 | 0.31 | 11.8 | 7.3 | -6.8 | 120 | 0.282849 | 0.000014 | 2.74 | 5.87 | 0.26 |
| T9'-12@11.ais | 4.49 | 0.10 | 1.56 | 0.23 | 30.0 | 9.9 | -9.0 | 449 | - | - | - | - | - |
| TVG-C1@20 | - | - | - | - | - | - | - | - | 0.282766 | 0.000049 | -0.20 | 6.48 | 0.10 |

Note: “–“ means not collected.

**Table S2. SIMS U-Pb dating, O isotopes and LA-MC-ICP-MS Lu-Hf isotope results for the inherited zircon cores in the back-arc volcanic rocks.**

|  | **^U-Th-Pb isotope ratios^** | | | | | | **^Ages (Myr)^** | | | | | |  |  |  |  |  |  |  |  |  |
| --- | --- | --- | --- | --- | --- | --- | --- | --- | --- | --- | --- | --- | --- | --- | --- | --- | --- | --- | --- | --- | --- |
| ^207^Pb/^235^U | ^±σ%^ | ^206^Pb/^238^U | ^±σ%^ | ^207^Pb/^206^Pb | ^±σ%^ | ^207^Pb/^206^Pb | ^±σ%^ | ^207^Pb/^235^U | ^±σ%^ | ^206^Pb/^238^U | ^±σ%^ | U (ppm) | Th/U | ^176^Hf/^177^Hf | 2SE | εHf _(0)_ | εHf _(t)_ | T_DM2_ | δ^18^O | 2SE |  |
| **C1@05** | 1.11193 | 2.88 | 0.1219 | 1.53 | 0.06613 | 2.44 | 810.6 | 50.2 | 759.1 | 15.5 | **741.7** | 10.7 | 243 | 0.88 | 0.281885 | 0.000014 | -31.4 | -15.7 | 2635 | 5.76 | 0.13 |
| **C1@09** | 1.67616 | 1.72 | 0.1672 | 1.55 | 0.07271 | 0.73 | **1005.9** | 14.8 | 999.5 | 11.0 | 996.6 | 14.3 | 843 | 0.45 | 0.282030 | 0.000012 | -26.2 | -4.9 | 2154 | 7.98 | 0.11 |
| **C1@20** | 4.86361 | 1.62 | 0.3086 | 1.50 | 0.11431 | 0.61 | **1869.1** | 11.0 | 1796.0 | 13.7 | 1733.7 | 22.8 | 542 | 0.33 | - | - | - | - | - | 10.66 | 0.07 |
| **C1@08** | 11.56759 | 1.60 | 0.4598 | 1.52 | 0.18245 | 0.49 | **2675.3** | 8.1 | 2570.1 | 15.0 | 2438.8 | 30.9 | 395 | 0.14 | 0.281232 | 0.000015 | -54.5 | 4.9 | 2837 | 5.74 | 0.15 |
| **C1@07** | 12.41889 | 2.52 | 0.5039 | 2.19 | 0.17875 | 1.25 | **2641.3** | 20.5 | 2636.6 | 24.0 | 2630.4 | 47.5 | 227 | 0.40 | 0.281244 | 0.000016 | -54.0 | 4.7 | 2825 | 5.85 | 0.08 |
| **C1-2@08** | 7.13741 | 2.20 | 0.3549 | 1.95 | 0.14587 | 1.02 | **2298.1** | 17.3 | 2128.7 | 19.8 | 1957.8 | 33.1 | 1042 | 0.22 | - | - | - | - | - | 6.93 | 0.22 |
| **C1-2@12** | 5.68635 | 1.57 | 0.3295 | 1.50 | 0.12516 | 0.47 | **2031.1** | 8.3 | 1929.3 | 13.7 | 1836.0 | 24.0 | 756 | 0.44 | - | - | - | - | - | 8.53 | 0.28 |
| **C1-2@15** | 1.84593 | 1.58 | 0.1657 | 1.50 | 0.08081 | 0.49 | **1216.8** | 9.7 | 1062.0 | 10.5 | 988.2 | 13.8 | 1141 | 0.46 | - | - | - | - | - | 4.49 | 0.21 |
| **C1-1@05** | 5.28034 | 1.61 | 0.3233 | 1.50 | 0.11846 | 0.58 | **1933.1** | 10.4 | 1865.7 | 13.9 | 1805.8 | 23.7 | 1100 | 0.04 | - | - | - | - | - | 6.99 | 0.26 |
| **C1-1@07** | 8.65435 | 1.78 | 0.3813 | 1.52 | 0.16460 | 0.92 | **2503.5** | 15.4 | 2302.3 | 16.3 | 2082.5 | 27.1 | 172 | 0.82 | 0.281304 | 0.000013 | -51.9 | 3.5 | 2790 | 8.26 | 0.14 |
| **C1-1@15** | 7.70496 | 1.57 | 0.3711 | 1.50 | 0.15059 | 0.46 | **2352.6** | 7.8 | 2197.2 | 14.2 | 2034.5 | 26.2 | 1387 | 0.32 | - | - | - | - | - | 7.17 | 0.25 |
| **T9**'**@18** | 9.13053 | 1.57 | 0.3946 | 1.51 | 0.16784 | 0.44 | **2536.2** | 7.3 | 2351.2 | 14.5 | 2143.9 | 27.6 | 273 | 0.88 | - | - | - | - | - | 5.89 | 0.16 |
| **C1@15** | 0.15382 | 3.61 | 0.0230 | 1.65 | 0.04843 | 3.21 | 120.6 | 74.0 | 145.3 | 4.9 | **146.8** | 2.4 | 1203 | 2.54 | - | - | - | - | - | 5.83 | 0.09 |
| **C1@02** | 0.16207 | 2.37 | 0.0237 | 1.52 | 0.04964 | 1.82 | 178.1 | 41.9 | 152.5 | 3.4 | **150.9** | 2.3 | 1636 | 0.46 | 0.282554 | 0.000015 | -7.7 | -4.6 | 1494 | 5.33 | 0.13 |
| **C1@03** | 0.16301 | 2.38 | 0.0246 | 1.50 | 0.04815 | 1.85 | 106.6 | 43.1 | 153.3 | 3.4 | **156.4** | 2.3 | 1110 | 0.35 | - | - | - | - | - | 5.59 | 0.16 |
| **C1@11** | 0.16186 | 5.14 | 0.0250 | 1.66 | 0.04699 | 4.86 | 48.8 | 112.1 | 152.3 | 7.3 | **159.1** | 2.6 | 765 | 0.47 | - | - | - | - | - | - | - |
| **C1-2@05** | 0.26698 | 1.84 | 0.0375 | 1.50 | 0.05159 | 1.07 | 267.1 | 24.3 | 240.3 | 3.9 | **237.5** | 3.5 | 745 | 0.51 | 0.282467 | 0.000017 | -10.8 | -5.7 | 1632 | 8.91 | 0.12 |
| **C1-1@14** | 0.15115 | 7.51 | 0.0253 | 1.57 | 0.04328 | 7.35 | -151.4 | 173.0 | 142.9 | 10.1 | **161.2** | 2.5 | 446 | 0.44 | 0.282581 | 0.000022 | -6.8 | -3.3 | 1419 | 8.30 | 0.27 |
| **C1-1@17** | 0.45658 | 1.82 | 0.0592 | 1.50 | 0.05594 | 1.03 | 450.0 | 22.7 | 381.9 | 5.8 | **370.7** | 5.4 | 2384 | 0.71 | - | - | - | - | - | 6.34 | 0.20 |
| **R10-H2@04** | 0.11074 | 2.47 | 0.0169 | 1.51 | 0.04745 | 1.95 | 72.0 | 45.8 | 106.6 | 2.5 | **108.2** | 1.6 | 1415 | 1.01 | - | - | - | - | - | - | - |

Note: The εHf_(0)_, εHf_(t)_, and T_DM2_ were calculated following the methods of Griffin et al.^23^; “–“ denotes not collected.

**Table S3. SIMS trace element results for the Light-CL zircons and their inherited cores in the volcanic rocks (ppm).**

|  | La | Ce | Pr | Nd | Sm | Eu | Gd | Tb | Dy | Ho | Er | Tm | Yb | Lu | Y | Th | U | Hf | Th/U | ^a^Ti | ^b^T (℃) |
| --- | --- | --- | --- | --- | --- | --- | --- | --- | --- | --- | --- | --- | --- | --- | --- | --- | --- | --- | --- | --- | --- |
| C1-1@1 | 0.06 | 7.47 | 0.03 | 0.61 | 1.71 | 0.31 | 10.98 | 4.69 | 59 | 26 | 126 | 28 | 266 | 60 | 778 | 63 | 129 | 11425 | 0.49 | 6.21 | 701 |
| C1-2@2 | 0.19 | 9.34 | 0.16 | 2.15 | 4.86 | 0.79 | 27.63 | 11.63 | 141 | 60 | 266 | 57 | 515 | 115 | 1709 | 125 | 185 | 11199 | 0.68 | 5.08 | 685 |
| C1-3@3 | 0.06 | 6.82 | 0.02 | 0.52 | 1.51 | 0.32 | 10.30 | 4.43 | 58 | 26 | 121 | 27 | 259 | 59 | 750 | 57 | 113 | 11939 | 0.50 | 5.28 | 688 |
| C1-4@4 | 0.06 | 7.90 | 0.09 | 2.09 | 4.72 | 0.71 | 25.80 | 10.23 | 128 | 53 | 237 | 51 | 460 | 103 | 1541 | 101 | 149 | 11034 | 0.67 | 5.36 | 689 |
| C1-6@5 | 1.36 | 18.38 | 0.67 | 6.20 | 8.06 | 0.77 | 43.31 | 16.20 | 183 | 70 | 297 | 62 | 528 | 113 | 2163 | 335 | 688 | 10883 | 0.49 | 3.85 | 663 |
| C1-5@6 | 0.16 | 13.98 | 0.15 | 2.05 | 4.75 | 0.86 | 29.49 | 13.14 | 168 | 74 | 337 | 75 | 681 | 158 | 2161 | 212 | 283 | 10163 | 0.75 | 8.77 | 729 |
| C1-7@7 | 0.04 | 7.82 | 0.04 | 0.65 | 2.03 | 0.33 | 12.59 | 5.71 | 75 | 33 | 157 | 36 | 333 | 80 | 1002 | 71 | 139 | 10568 | 0.51 | 5.79 | 695 |
| C1-8@8 | 0.22 | 8.04 | 0.08 | 1.16 | 2.41 | 0.48 | 15.43 | 6.99 | 88 | 39 | 186 | 42 | 390 | 93 | 1211 | 66 | 131 | 10300 | 0.50 | 6.32 | 702 |
| **^c^**C1-9@9 | 0.07 | 6.45 | 0.04 | 0.37 | 1.30 | 0.23 | 8.07 | 3.62 | 47 | 21 | 98 | 23 | 204 | 50 | 640 | 44 | 103 | 11384 | 0.43 | 6.31 | 702 |
| **^d^**C1-10@10 | 0.06 | 9.29 | 0.20 | 2.84 | 2.73 | 0.95 | 9.30 | 3.15 | 33 | 13 | 57 | 13 | 122 | 31 | 424 | 59 | 99 | 9731 | 0.59 | 3.28 | 651 |
| C1-11@11 | 1.51 | 18.90 | 0.36 | 2.29 | 2.48 | 0.46 | 16.60 | 8.53 | 124 | 63 | 342 | 87 | 914 | 225 | 1956 | 377 | 977 | 11130 | 0.39 | 34.83 | 864 |
| C1-12@12 | 0.14 | 16.70 | 0.24 | 4.16 | 8.27 | 1.45 | 45.58 | 18.85 | 224 | 92 | 405 | 86 | 757 | 168 | 2679 | 336 | 325 | 10931 | 1.03 | 6.53 | 705 |
| C1-13@13 | 0.09 | 21.77 | 0.35 | 6.66 | 14.49 | 3.11 | 80.87 | 33.26 | 390 | 156 | 671 | 138 | 1235 | 261 | 4270 | 462 | 472 | 10451 | 0.98 | 5.92 | 697 |
| C1-14@14 | 0.77 | 9.70 | 0.25 | 2.15 | 3.33 | 0.74 | 13.44 | 4.37 | 43 | 15 | 66 | 14 | 122 | 29 | 491 | 102 | 364 | 10382 | 0.28 | 24.08 | 824 |
| C1-15@15 | 0.09 | 7.06 | 0.04 | 0.68 | 1.43 | 0.24 | 9.46 | 4.28 | 53 | 23 | 109 | 25 | 227 | 53 | 733 | 64 | 126 | 10554 | 0.51 | 5.48 | 691 |
| C1-1-1@1 | 2.75 | 26.03 | 0.96 | 4.77 | 3.57 | 0.47 | 16.14 | 5.83 | 60 | 24 | 102 | 23 | 195 | 45 | 762 | 84 | 110 | 11202 | 0.76 | 33.30 | 859 |
| C1-1-2@2 | 0.67 | 22.41 | 0.25 | 2.14 | 1.63 | 0.46 | 8.60 | 3.18 | 33 | 13 | 53 | 13 | 118 | 28 | 395 | 82 | 160 | 12482 | 0.51 | 14.06 | 772 |
| C1-1-3@3 | 0.93 | 6.80 | 0.35 | 1.57 | 1.71 | 0.34 | 10.69 | 4.55 | 55 | 21 | 107 | 27 | 257 | 63 | 700 | 39 | 481 | 11942 | 0.08 | 19.81 | 805 |
| C1-1-4@4 | 0.60 | 13.48 | 0.28 | 1.63 | 1.48 | 0.39 | 9.03 | 4.01 | 47 | 20 | 96 | 23 | 213 | 51 | 627 | 107 | 434 | 12016 | 0.25 | 16.51 | 787 |
| C1-1-5@5 | 2.28 | 12.36 | 0.63 | 3.03 | 2.29 | 0.36 | 11.77 | 5.76 | 68 | 30 | 139 | 32 | 284 | 68 | 912 | 66 | 121 | 10329 | 0.54 | 16.68 | 788 |
| C1-1-6@6 | 8.97 | 78.13 | 3.40 | 20.64 | 15.39 | 2.23 | 59.17 | 22.47 | 265 | 107 | 487 | 107 | 959 | 210 | 3228 | 770 | 1089 | 10714 | 0.71 | 444.85 | 1238 |
| C1-1-7@7 | 0.32 | 8.53 | 0.16 | 1.07 | 2.12 | 0.34 | 12.58 | 5.47 | 71 | 30 | 142 | 34 | 301 | 70 | 908 | 80 | 149 | 10573 | 0.53 | 17.99 | 795 |
| C1-1-8@8 | 8.15 | 24.00 | 2.17 | 10.15 | 5.67 | 0.66 | 18.66 | 6.75 | 73 | 30 | 126 | 27 | 235 | 52 | 882 | 132 | 315 | 9441 | 0.42 | 97.33 | 990 |
| C1-1-9@9 | 0.52 | 37.20 | 0.33 | 2.99 | 5.61 | 1.32 | 22.78 | 8.25 | 88 | 30 | 125 | 27 | 226 | 49 | 950 | 176 | 660 | 9515 | 0.27 | 12.25 | 759 |
| ***Table S3 (continued)*** | | | | | | | | | | | | |  |  |  |  |  |  |  |  |  |
| C1-1-10@10 | 0.13 | 13.09 | 0.23 | 3.71 | 7.41 | 1.23 | 40.21 | 16.88 | 200 | 80 | 350 | 77 | 666 | 151 | 2368 | 176 | 222 | 9789 | 0.79 | 7.22 | 713 |
| C1-1-11@11 | 2.30 | 13.31 | 0.56 | 2.55 | 2.05 | 0.42 | 11.13 | 4.75 | 64 | 27 | 125 | 30 | 264 | 63 | 821 | 62 | 105 | 9944 | 0.59 | 47.96 | 900 |
| C1-2-9@1 | 0.21 | 8.20 | 0.07 | 0.93 | 2.09 | 0.47 | 14.43 | 6.14 | 80 | 33 | 160 | 38 | 337 | 79 | 1048 | 81 | 170 | 10965 | 0.47 | 13.11 | 765 |
| C1-2-8@2 | 0.45 | 7.59 | 0.15 | 0.80 | 1.63 | 0.33 | 10.82 | 4.77 | 58 | 26 | 121 | 28 | 261 | 62 | 791 | 45 | 98 | 10425 | 0.46 | 11.26 | 751 |
| C1-2-7@3 | 0.71 | 10.17 | 0.25 | 1.02 | 2.04 | 0.52 | 12.75 | 5.68 | 70 | 31 | 143 | 32 | 291 | 70 | 912 | 118 | 177 | 10469 | 0.67 | 23.33 | 821 |
| C1-2-6@4 | 0.38 | 8.12 | 0.16 | 1.48 | 3.04 | 0.52 | 17.78 | 7.71 | 95 | 41 | 189 | 42 | 379 | 89 | 1234 | 67 | 122 | 10344 | 0.55 | 8.04 | 722 |
| C1-2-5@5 | 1.91 | 11.19 | 0.79 | 5.02 | 4.07 | 0.56 | 16.15 | 6.25 | 76 | 33 | 156 | 37 | 328 | 80 | 1024 | 65 | 123 | 9500 | 0.53 | 44.62 | 892 |
| C1-2-4@6 | 0.56 | 10.73 | 0.35 | 3.96 | 4.97 | 0.73 | 27.63 | 10.55 | 123 | 49 | 222 | 47 | 436 | 98 | 1490 | 195 | 592 | 10891 | 0.33 | 8.79 | 730 |
| C1-2-3@7 | 45.37 | 308.62 | 55.90 | 313.19 | 172.04 | 51.27 | 211.60 | 55.61 | 443 | 117 | 439 | 96 | 865 | 182 | 3631 | 274 | 2261 | 19873 | 0.12 | 26.09 | 833 |
| C1-2-2@8 | 4.85 | 46.33 | 2.78 | 16.53 | 30.56 | 18.83 | 148.93 | 51.92 | 382 | 105 | 389 | 75 | 644 | 140 | 3082 | 359 | 419 | 11683 | 0.86 | 387.25 | 1211 |
| C1-2-1@9 | 0.45 | 14.62 | 0.49 | 3.73 | 5.76 | 1.29 | 30.35 | 11.45 | 138 | 54 | 245 | 52 | 457 | 99 | 1579 | 201 | 658 | 14198 | 0.31 | 8.25 | 724 |
| T9'-2@2 | 1.40 | 14.71 | 2.10 | 18.27 | 18.07 | 3.74 | 64.39 | 21.84 | 217 | 75 | 300 | 63 | 513 | 113 | 2250 | 229 | 502 | 9389 | 0.46 | 7.95 | 721 |
| T9'-3@3 | 16.79 | 90.90 | 15.72 | 92.98 | 58.39 | 19.80 | 115 | 36.94 | 333 | 112 | 500 | 124 | 1197 | 268 | 3604 | 169 | 809 | 12452 | 0.21 | 1553 | 1529 |
| T9'-4@4 | 1.08 | 74.37 | 0.68 | 6.88 | 8.26 | 3.41 | 29.83 | 10.43 | 109 | 42 | 192 | 43 | 391 | 96 | 1382 | 375 | 411 | 9648 | 0.91 | 27.69 | 839 |
| T9'-5@5 | 1.61 | 25.83 | 0.84 | 5.75 | 7.51 | 4.10 | 34.86 | 14.94 | 148 | 55 | 232 | 56 | 527 | 123 | 1648 | 402 | 953 | 12060 | 0.42 | 24.74 | 827 |
| T9'-6@6 | 7.03 | 43.66 | 2.81 | 17.53 | 17.77 | 9.92 | 70.67 | 23.03 | 207 | 59 | 206 | 39 | 337 | 76 | 1879 | 128 | 200 | 11646 | 0.64 | 93.94 | 985 |
| T9'-7@7 | 0.46 | 12.02 | 0.49 | 5.06 | 6.89 | 1.60 | 30.68 | 11.14 | 122 | 49 | 213 | 46 | 413 | 98 | 1487 | 134 | 173 | 9308 | 0.77 | 9.58 | 737 |
| T9'-8@8 | 1.00 | 113.69 | 0.96 | 6.35 | 7.48 | 2.12 | 31.71 | 11.32 | 130 | 54 | 253 | 58 | 533 | 124 | 1761 | 477 | 383 | 10899 | 1.24 | 24.38 | 826 |
| T9'-9@9 | 0.14 | 7.66 | 0.16 | 2.33 | 4.75 | 0.75 | 27.84 | 11.87 | 142 | 61 | 276 | 61 | 534 | 122 | 1780 | 131 | 228 | 10462 | 0.57 | 5.98 | 698 |
| T9'-10@10 | 0.15 | 10.38 | 0.17 | 3.21 | 7.40 | 1.16 | 41.25 | 17.66 | 215 | 89 | 399 | 88 | 737 | 169 | 2573 | 221 | 334 | 10677 | 0.66 | 21.12 | 811 |
| T9'-11@11 | 0.46 | 13.45 | 0.18 | 1.32 | 3.19 | 0.55 | 21.68 | 9.65 | 129 | 55 | 258 | 58 | 529 | 121 | 1601 | 320 | 414 | 10632 | 0.77 | 23.21 | 821 |
| T9'-12@12 | 0.76 | 13.11 | 0.19 | 1.78 | 2.81 | 0.44 | 19.63 | 8.56 | 113 | 52 | 250 | 59 | 536 | 131 | 1589 | 144 | 310 | 10492 | 0.46 | 41.03 | 882 |
| T9'-13@13 | 1.52 | 14.09 | 0.30 | 2.12 | 2.76 | 0.53 | 18.46 | 8.78 | 116 | 54 | 261 | 63 | 583 | 138 | 1640 | 147 | 307 | 9863 | 0.48 | 30.25 | 848 |
| T9'-14@14 | 3.31 | 20.19 | 1.00 | 4.97 | 4.24 | 0.59 | 22.27 | 9.80 | 128 | 60 | 286 | 65 | 605 | 144 | 1754 | 194 | 356 | 11002 | 0.55 | 23.28 | 821 |
| T9'-15@15 | 0.45 | 14.19 | 0.24 | 2.50 | 4.92 | 0.78 | 31.25 | 13.52 | 169 | 76 | 350 | 79 | 710 | 165 | 2199 | 228 | 379 | 10684 | 0.60 | 10.55 | 746 |
| ***Table S3 (continued)*** | | | |  |  |  |  |  |  |  |  |  |  |  |  |  |  |  |  |  |  |
| T9'-16@16 | 1.47 | 14.91 | 0.68 | 4.67 | 5.83 | 0.64 | 32.67 | 13.91 | 171 | 72 | 334 | 73 | 639 | 150 | 2123 | 214 | 348 | 11054 | 0.61 | 3.87 | 664 |
| T9'-17@17 | 0.15 | 11.16 | 0.29 | 3.61 | 7.98 | 1.18 | 45.44 | 18.92 | 234 | 97 | 429 | 92 | 798 | 183 | 2807 | 242 | 332 | 10351 | 0.73 | 19.89 | 805 |
| T9'-18@18 | 1.26 | 30.30 | 0.36 | 2.12 | 2.28 | 0.81 | 14.45 | 5.65 | 75 | 32 | 166 | 40 | 377 | 97 | 1042 | 166 | 330 | 10876 | 0.50 | 17.91 | 795 |
| T9'-19@19 | 0.69 | 13.80 | 0.19 | 2.08 | 4.72 | 0.52 | 27.58 | 11.62 | 153 | 66 | 309 | 70 | 618 | 149 | 1943 | 243 | 373 | 10699 | 0.65 | 22.91 | 819 |
| T9'-20@20 | 0.47 | 8.95 | 0.10 | 1.17 | 2.08 | 0.45 | 13.29 | 6.45 | 82 | 39 | 195 | 48 | 445 | 111 | 1202 | 85 | 207 | 11320 | 0.41 | 110.71 | 1008 |
| T9'-21@21 | 0.14 | 8.67 | 0.11 | 1.26 | 2.70 | 0.36 | 19.19 | 8.19 | 110 | 47 | 224 | 49 | 456 | 109 | 1410 | 116 | 231 | 10884 | 0.50 | 13.23 | 766 |
| T9'-22@22 | 3.07 | 13.11 | 0.68 | 3.61 | 2.97 | 0.71 | 17.21 | 7.85 | 96 | 43 | 205 | 47 | 428 | 102 | 1289 | 99 | 205 | 8154 | 0.48 | 84.70 | 971 |
| T9'-23@23 | 0.37 | 12.77 | 0.16 | 2.30 | 4.44 | 0.82 | 30.63 | 13.38 | 170 | 75 | 355 | 83 | 721 | 171 | 2229 | 185 | 345 | 10499 | 0.54 | 24.76 | 827 |
| T9'-24@24 | 2.84 | 93.82 | 1.41 | 18.68 | 34.64 | 3.80 | 197.81 | 77.17 | 906 | 362 | 1571 | 327 | 2676 | 577 | 10360 | 2278 | 1807 | 7754 | 1.26 | 37.44 | 872 |
| R10-H2-1@1 | 0.39 | 9.48 | 0.24 | 2.17 | 4.73 | 0.78 | 31.98 | 13.20 | 158 | 67 | 316 | 67 | 574 | 133 | 1943 | 160 | 253 | 10262 | 0.63 | 12.40 | 760 |
| R10-H2-2@2 | 0.58 | 12.28 | 0.23 | 2.97 | 6.45 | 1.36 | 42.59 | 17.29 | 208 | 88 | 401 | 87 | 752 | 171 | 2562 | 231 | 339 | 9789 | 0.68 | 13.91 | 771 |
| R10-H2-3@3 | 3.16 | 21.66 | 0.81 | 5.41 | 4.65 | 0.57 | 24.24 | 10.47 | 135 | 59 | 275 | 61 | 581 | 124 | 1690 | 288 | 414 | 10514 | 0.70 | 45.50 | 894 |
| R10-H2-4@4 | 0.44 | 13.80 | 0.15 | 1.59 | 3.32 | 0.52 | 22.45 | 9.53 | 128 | 56 | 264 | 63 | 555 | 132 | 1646 | 231 | 392 | 11172 | 0.59 | 10.13 | 742 |
| R10-H2-5@5 | 3.25 | 15.13 | 1.19 | 5.41 | 3.95 | 0.52 | 19.17 | 7.78 | 101 | 42 | 192 | 45 | 386 | 92 | 1232 | 103 | 176 | 11069 | 0.58 | 5.83 | 696 |
| R10-H2-6@6 | 0.67 | 35.00 | 0.63 | 6.66 | 13.65 | 2.28 | 69.49 | 28.15 | 338 | 143 | 636 | 139 | 1237 | 274 | 4264 | 795 | 826 | 10832 | 0.96 | 8.06 | 722 |
| R10-H2-7@7 | 0.77 | 16.20 | 0.26 | 3.55 | 6.51 | 1.37 | 43.63 | 17.54 | 208 | 89 | 391 | 85 | 750 | 169 | 2582 | 338 | 422 | 10159 | 0.80 | 46.73 | 897 |
| R10-H2-8@8 | 0.25 | 9.38 | 0.11 | 0.88 | 2.80 | 0.39 | 14.55 | 6.68 | 87 | 38 | 177 | 39 | 359 | 85 | 1123 | 131 | 233 | 10610 | 0.56 | 12.67 | 762 |
| R10-H2-9@9 | 0.16 | 9.54 | 0.16 | 1.53 | 3.47 | 0.61 | 24.47 | 9.91 | 125 | 53 | 259 | 56 | 511 | 122 | 1609 | 128 | 250 | 10891 | 0.51 | 11.66 | 755 |
| R10-H2-10@10 | 0.58 | 15.81 | 0.38 | 3.07 | 5.92 | 0.82 | 36.57 | 15.29 | 199 | 83 | 369 | 84 | 718 | 163 | 2383 | 305 | 420 | 10617 | 0.72 | 7.98 | 721 |
| R10-H2-11@11 | 0.26 | 12.55 | 0.17 | 2.75 | 5.93 | 0.63 | 34.17 | 14.91 | 179 | 77 | 344 | 75 | 665 | 145 | 2170 | 240 | 386 | 11379 | 0.62 | 11.21 | 751 |
| R10-H2-12@12 | 15.19 | 51.02 | 4.88 | 26.87 | 11.03 | 1.42 | 47.92 | 18.78 | 221 | 91 | 411 | 90 | 766 | 177 | 2668 | 305 | 445 | 10284 | 0.69 | 6.84 | 709 |
| R10-H2-13@13 | 0.18 | 4.30 | 0.16 | 2.84 | 6.04 | 0.39 | 38.28 | 15.38 | 182 | 70 | 302 | 62 | 540 | 115 | 2125 | 170 | 319 | 11624 | 0.53 | 11.46 | 753 |
| R10-H2-14@14 | 0.15 | 14.96 | 0.29 | 3.65 | 6.72 | 1.08 | 40.37 | 16.93 | 214 | 91 | 406 | 92 | 836 | 182 | 2593 | 316 | 431 | 10307 | 0.73 | 10.92 | 749 |
| R10-H2-15@15 | 0.48 | 11.77 | 0.17 | 1.13 | 2.51 | 0.54 | 17.65 | 8.59 | 108 | 49 | 237 | 55 | 502 | 116 | 1465 | 208 | 298 | 10351 | 0.70 | 17.90 | 795 |
| R10-H2-16@16 | 1.32 | 11.89 | 0.46 | 2.22 | 4.11 | 0.51 | 25.62 | 10.09 | 135 | 57 | 269 | 60 | 531 | 122 | 1711 | 183 | 302 | 10025 | 0.60 | 28.45 | 842 |
| ***Table S3 (continued)*** | | |  |  |  |  |  |  |  |  |  |  |  |  |  |  |  |  |  |  |  |
| R10-H2-17@17 | 0.43 | 8.59 | 0.15 | 1.18 | 2.31 | 0.38 | 13.73 | 5.83 | 83 | 38 | 185 | 43 | 400 | 93 | 1144 | 111 | 278 | 10691 | 0.40 | 14.35 | 774 |
| R10-H2-18@18 | 1.10 | 12.41 | 0.35 | 3.54 | 4.22 | 0.80 | 26.87 | 11.44 | 147 | 65 | 289 | 65 | 582 | 133 | 1866 | 175 | 304 | 10543 | 0.58 | 17.09 | 790 |
| R10-H2-19@19 | 0.30 | 8.04 | 0.13 | 1.18 | 3.77 | 0.56 | 20.38 | 8.86 | 115 | 50 | 237 | 51 | 468 | 105 | 1457 | 121 | 221 | 11061 | 0.55 | 5.64 | 693 |
| R10-H2-20@20 | 0.51 | 10.20 | 0.20 | 1.36 | 2.22 | 0.45 | 14.36 | 5.90 | 82 | 36 | 181 | 42 | 396 | 99 | 1126 | 113 | 237 | 10810 | 0.48 | 24.63 | 827 |
| R10-H2-21@21 | 1.20 | 11.47 | 0.25 | 1.95 | 3.08 | 0.65 | 19.28 | 8.43 | 114 | 49 | 226 | 55 | 486 | 114 | 1504 | 194 | 300 | 10092 | 0.65 | 58.57 | 924 |
| R10-H2-22@22 | 0.18 | 14.99 | 0.20 | 2.67 | 5.94 | 0.85 | 35.84 | 15.18 | 195 | 85 | 391 | 85 | 763 | 173 | 2490 | 264 | 408 | 10181 | 0.65 | 7.75 | 719 |
| R10-H2-23@23 | 2.67 | 19.58 | 0.80 | 5.49 | 6.02 | 0.74 | 29.00 | 12.59 | 155 | 69 | 315 | 68 | 617 | 141 | 2014 | 237 | 370 | 10529 | 0.64 | 10.57 | 746 |

Note: a) Ti _91500_ corrected; b) T (℃) a_TiO2_=1; c) C1-9@9 and C1-10@10 are the results for the representative Light-CL zircon rim and its inherited core, respectively, as shown in Fig. 3.

**Table S4.** Sampling locations of the studied volcanic rocks from the Okinawa Trough.

| **Sample** | **Latitude, N** | **Longitude, E** | **Depth, m** | **Rock-type** |
| --- | --- | --- | --- | --- |
| **C1** | 24°54’07.027″ | 122°32’33.783″ | 1176 | Dacite |
| **T9’** | 24˚50´57.774´´ | 122˚41´55.877´´ | 1382 | Rhyolite |
| **R10-H2** | 24°50’47.876´´ | 122°42’01.783´´ | 1378 | Rhyolite |

**Table S5.** U-Th isotope compositions for the reference AS3 and Qinghu zircon standards.

| **Standards** | **(^230^Th/^232^Th)** | **1s** | **(^238^U/^232^Th)** | **1s** | **(^230^Th/^238^U)** | **1s** |
| --- | --- | --- | --- | --- | --- | --- |
| as3@1.ais | 2.980 | 0.039 | 2.635 | 0.037 | 1.131 | 0.022 |
| as3@2.ais | 2.964 | 0.049 | 2.706 | 0.106 | 1.095 | 0.047 |
| as3@3.ais | 3.030 | 0.055 | 2.749 | 0.037 | 1.102 | 0.025 |
| as3@4.ais | 4.991 | 0.121 | 4.864 | 0.065 | 1.026 | 0.028 |
| as3@5.ais | 4.282 | 0.082 | 4.187 | 0.083 | 1.023 | 0.028 |
| as3@6.ais | 4.569 | 0.175 | 4.002 | 0.079 | 1.142 | 0.049 |
| qinghu@1.ais | 5.272 | 0.148 | 5.054 | 0.103 | 1.043 | 0.036 |
| qinghu@2.ais | 14.168 | 0.381 | 13.061 | 0.378 | 1.085 | 0.043 |
| qinghu@3.ais | 5.021 | 0.210 | 5.013 | 0.099 | 1.002 | 0.046 |
| qinghu@4.ais | 5.877 | 0.181 | 5.672 | 0.127 | 1.036 | 0.039 |
| qinghu@5.ais | 5.837 | 0.206 | 5.329 | 0.107 | 1.095 | 0.044 |
| qinghu@6.ais | 5.648 | 0.261 | 5.156 | 0.106 | 1.095 | 0.055 |
| qinghu@7.ais | 7.662 | 0.269 | 6.472 | 0.129 | 1.184 | 0.048 |
| qinghu@8.ais | 7.804 | 0.373 | 7.386 | 0.148 | 1.057 | 0.055 |
| qinghu@9.ais | 6.740 | 0.370 | 5.687 | 0.113 | 1.185 | 0.069 |
| qinghu@10.ais | 7.232 | 0.354 | 6.041 | 0.120 | 1.197 | 0.063 |
| qinghu@11.ais | 7.635 | 0.194 | 7.160 | 0.142 | 1.066 | 0.034 |
| qinghu@12.ais | 7.442 | 0.188 | 6.832 | 0.135 | 1.089 | 0.035 |
| qinghu@13.ais | 5.970 | 0.205 | 5.777 | 0.137 | 1.033 | 0.043 |
| qinghu@14.ais | 6.819 | 0.463 | 6.785 | 0.135 | 1.005 | 0.071 |
| qinghu@15.ais | 9.517 | 0.268 | 8.646 | 0.172 | 1.101 | 0.038 |
| qinghu@16.ais | 7.821 | 0.282 | 7.509 | 0.152 | 1.042 | 0.043 |
| qinghu@17.ais | 6.576 | 0.180 | 6.372 | 0.126 | 1.032 | 0.035 |
| qinghu@18.ais | 5.317 | 0.171 | 4.837 | 0.098 | 1.099 | 0.042 |
| qinghu@19.ais | 7.502 | 0.234 | 7.122 | 0.141 | 1.053 | 0.039 |
| qinghu@20.ais | 6.228 | 0.239 | 5.578 | 0.129 | 1.116 | 0.050 |
| qinghu@21.ais | 6.009 | 0.147 | 5.769 | 0.118 | 1.042 | 0.033 |
| qinghu@22.ais | 6.578 | 0.186 | 6.073 | 0.124 | 1.083 | 0.038 |
| qinghu@23.ais | 6.146 | 0.188 | 6.088 | 0.125 | 1.010 | 0.037 |
| qinghu@24.ais | 5.738 | 0.244 | 5.517 | 0.110 | 1.040 | 0.049 |
| qinghu@25.ais | 6.350 | 0.270 | 5.947 | 0.121 | 1.068 | 0.050 |

**Table S6.** U-Pb dating results for the Qinghu zircon standard measured via SIMS.

| **Sample/** | **[U]** | **[Th]** | **Th/U** | **f_206_%** | **^207^Pb** | **±** | **^206^Pb** | **±** | **^207^Pb** | **±** | **^t207/235^** | **±** | **^t206/238^** | **±** |
| --- | --- | --- | --- | --- | --- | --- | --- | --- | --- | --- | --- | --- | --- | --- |
| **spot #** | **ppm** | **ppm** | **meas** |  | **^235^U** | **%** | **^238^U** | **%** | **^206^Pb** | **%** | **^/Ma^** |  | **^/Ma^** |  |
| [Qinghu@1](mailto:Qinghu@5) | 772 | 337 | 0.44 | 0.54 | 0.1707 | 2.73 | 0.0251 | 1.64 | 0.0493 | 2.18 | 160.0 | 4.0 | 159.9 | 2.6 |
| Qinghu@2 | 982 | 456 | 0.46 | 0.27 | 0.1746 | 2.45 | 0.0253 | 1.57 | 0.0501 | 1.88 | 163.4 | 3.7 | 161.0 | 2.5 |
| Qinghu@3 | 1431 | 678 | 0.47 | 0.23 | 0.1720 | 2.41 | 0.0258 | 1.51 | 0.0484 | 1.87 | 161.2 | 3.6 | 164.0 | 2.4 |
| Qinghu@4 | 1909 | 1105 | 0.58 | 0.23 | 0.1698 | 2.07 | 0.0252 | 1.56 | 0.0489 | 1.36 | 159.3 | 3.1 | 160.5 | 2.5 |
| Qinghu@5 | 1119 | 500 | 0.45 | 0.32 | 0.1608 | 3.21 | 0.0242 | 1.68 | 0.0482 | 2.73 | 151.4 | 4.5 | 154.2 | 2.6 |
| Qinghu@6 | 1108 | 506 | 0.46 | 0.15 | 0.1656 | 2.49 | 0.0245 | 1.53 | 0.0490 | 1.97 | 155.6 | 3.6 | 156.0 | 2.4 |
| Qinghu@7 | 1018 | 606 | 0.59 | 0.27 | 0.1713 | 2.66 | 0.0250 | 1.54 | 0.0497 | 2.17 | 160.5 | 4.0 | 159.0 | 2.4 |
| Qinghu@8 | 3216 | 2157 | 0.67 | 0.07 | 0.1709 | 2.30 | 0.0251 | 1.87 | 0.0494 | 1.34 | 160.2 | 3.4 | 159.9 | 3.0 |
| Qinghu@9 | 1607 | 728 | 0.45 | 0.30 | 0.1717 | 2.06 | 0.0253 | 1.53 | 0.0492 | 1.38 | 160.9 | 3.1 | 161.2 | 2.4 |
| Qinghu@10 | 997 | 385 | 0.39 | 0.07 | 0.1683 | 2.37 | 0.0248 | 1.50 | 0.0493 | 1.83 | 157.9 | 3.5 | 157.6 | 2.3 |
| Qinghu@11 | 1802 | 897 | 0.50 | 0.04 | 0.1702 | 2.00 | 0.0251 | 1.53 | 0.0492 | 1.29 | 159.6 | 3.0 | 159.7 | 2.4 |
| Qinghu@12 | 1465 | 626 | 0.43 | 0.10 | 0.1766 | 1.99 | 0.0258 | 1.51 | 0.0497 | 1.30 | 165.1 | 3.0 | 163.9 | 2.4 |
| Qinghu@13 | 756 | 231 | 0.31 | 0.09 | 0.1716 | 2.42 | 0.0254 | 1.56 | 0.0489 | 1.85 | 160.8 | 3.6 | 161.9 | 2.5 |
| Qinghu@14 | 1880 | 757 | 0.40 | 0.04 | 0.1708 | 1.99 | 0.0250 | 1.52 | 0.0495 | 1.30 | 160.1 | 3.0 | 159.2 | 2.4 |
| Qinghu@15 | 791 | 253 | 0.32 | 0.12 | 0.1689 | 2.37 | 0.0253 | 1.52 | 0.0484 | 1.82 | 158.4 | 3.5 | 161.1 | 2.4 |
| Qinghu@16 | 1170 | 472 | 0.40 | 0.27 | 0.1629 | 2.86 | 0.0247 | 1.52 | 0.0478 | 2.42 | 153.2 | 4.1 | 157.3 | 2.4 |
| Qinghu@17 | 1671 | 698 | 0.42 | 0.25 | 0.1642 | 2.66 | 0.0250 | 1.55 | 0.0476 | 2.16 | 154.4 | 3.8 | 159.3 | 2.4 |
| Qinghu@18 | 1031 | 435 | 0.42 | 0.16 | 0.1726 | 2.24 | 0.0254 | 1.52 | 0.0493 | 1.64 | 161.7 | 3.4 | 161.5 | 2.4 |
| Qinghu@19 | 3071 | 2066 | 0.67 | 0.01 | 0.1693 | 1.70 | 0.0249 | 1.67 | 0.0493 | 0.31 | 158.8 | 2.5 | 158.5 | 2.6 |
| Qinghu@20 | 1947 | 1043 | 0.54 | 0.13 | 0.1663 | 1.64 | 0.0245 | 1.56 | 0.0493 | 0.51 | 156.2 | 2.4 | 155.9 | 2.4 |
| Qinghu@21 | 1636 | 709 | 0.43 | 0.02 | 0.1708 | 1.56 | 0.0250 | 1.50 | 0.0495 | 0.42 | 160.1 | 2.3 | 159.3 | 2.4 |
| Qinghu@22 | 950 | 349 | 0.37 | 0.03 | 0.1723 | 1.63 | 0.0253 | 1.52 | 0.0495 | 0.57 | 161.4 | 2.4 | 160.8 | 2.4 |
| Qinghu@24 | 3071 | 2066 | 0.67 | 0.01 | 0.1693 | 1.70 | 0.0249 | 1.67 | 0.0493 | 0.31 | 158.8 | 2.5 | 158.5 | 2.6 |
| Qinghu@25 | 1947 | 1043 | 0.54 | 0.13 | 0.1663 | 1.64 | 0.0245 | 1.56 | 0.0493 | 0.51 | 156.2 | 2.4 | 155.9 | 2.4 |
| Qinghu@26 | 1636 | 709 | 0.43 | 0.02 | 0.1708 | 1.56 | 0.0250 | 1.50 | 0.0495 | 0.42 | 160.1 | 2.3 | 159.3 | 2.4 |
| Qinghu@27 | 950 | 349 | 0.37 | 0.03 | 0.1723 | 1.63 | 0.0253 | 1.52 | 0.0495 | 0.57 | 161.4 | 2.4 | 160.8 | 2.4 |
| Qinghu@28 | 1410 | 762 | 0.54 | 0.01 | 0.1672 | 1.83 | 0.0246 | 1.78 | 0.0493 | 0.45 | 157.0 | 2.7 | 156.7 | 2.7 |
| Qinghu@29 | 992 | 378 | 0.38 | 0.82 | 0.1707 | 2.56 | 0.0251 | 1.51 | 0.0493 | 2.07 | 160.1 | 3.8 | 159.9 | 2.4 |
| Qinghu@30 | 1283 | 458 | 0.36 | 0.02 | 0.1688 | 1.58 | 0.0251 | 1.50 | 0.0489 | 0.47 | 158.4 | 2.3 | 159.5 | 2.4 |
| Qinghu@31 | 1298 | 727 | 0.56 | 0.03 | 0.1717 | 1.65 | 0.0252 | 1.55 | 0.0494 | 0.56 | 160.9 | 2.5 | 160.6 | 2.5 |
| Qinghu@32 | 1906 | 1138 | 0.60 | 0.01 | 0.1711 | 1.60 | 0.0252 | 1.53 | 0.0493 | 0.46 | 160.4 | 2.4 | 160.1 | 2.4 |
| Qinghu@33 | 1930 | 985 | 0.51 | 0.02 | 0.1702 | 1.57 | 0.0252 | 1.51 | 0.0490 | 0.42 | 159.6 | 2.3 | 160.4 | 2.4 |
| Qinghu@34 | 1802 | 877 | 0.49 | 0.02 | 0.1675 | 1.56 | 0.0246 | 1.50 | 0.0493 | 0.42 | 157.2 | 2.3 | 156.9 | 2.3 |

**Table S7.** Oxygen isotope results for the Qinghu zircon standard measured by SIMS.

| **Sample** | **Intensity O^16^** | **O^16^/O^18^ Mean** | **δ^18^O** | **2SE** |
| --- | --- | --- | --- | --- |
| Qinghu@1 | 5.096E+9 | 2.022E-3 | 5.64 | 0.10 |
| Qinghu@2 | 5.018E+9 | 2.022E-3 | 5.61 | 0.10 |
| Qinghu@3 | 5.004E+9 | 2.021E-3 | 5.37 | 0.15 |
| Qinghu@4 | 4.997E+9 | 2.021E-3 | 5.34 | 0.13 |
| Qinghu@5 | 4.976E+9 | 2.021E-3 | 5.55 | 0.08 |
| Qinghu@6 | 4.958E+9 | 2.021E-3 | 5.36 | 0.28 |
| Qinghu@7 | 4.937E+9 | 2.022E-3 | 5.69 | 0.41 |
| Qinghu@9 | 4.616E+9 | 2.030E-3 | 5.81 | 0.14 |
| Qinghu@8 | 4.743E+9 | 2.029E-3 | 5.25 | 0.15 |
| Qinghu@7 | 4.813E+9 | 2.028E-3 | 5.74 | 0.11 |
| Qinghu@6 | 4.813E+9 | 2.027E-3 | 5.51 | 0.13 |
| Qinghu@5 | 4.889E+9 | 2.027E-3 | 5.33 | 0.10 |
| Qinghu@4 | 4.848E+9 | 2.027E-3 | 5.51 | 0.13 |
| Qinghu@3 | 4.887E+9 | 2.027E-3 | 5.25 | 0.09 |
| Qinghu@2 | 4.878E+9 | 2.026E-3 | 4.98 | 0.11 |
| Qinghu@10 | 4.611E+9 | 2.031E-3 | 5.92 | 0.10 |
| Qinghu@1 | 4.971E+9 | 2.026E-3 | 5.37 | 0.22 |
| Qinghu@1 | 1.638E+9 | 2.025E-3 | 5.53 | 0.23 |
| Qinghu@2 | 1.628E+9 | 2.025E-3 | 5.66 | 0.19 |
| Qinghu@3 | 1.638E+9 | 2.025E-3 | 5.77 | 0.29 |
| Qinghu@4 | 1.629E+9 | 2.025E-3 | 5.54 | 0.29 |
| Qinghu@5 | 1.639E+9 | 2.024E-3 | 5.36 | 0.30 |
| Qinghu@6 | 1.640E+9 | 2.025E-3 | 5.88 | 0.22 |
| Qinghu@7 | 1.632E+9 | 2.025E-3 | 5.59 | 0.22 |
| Qinghu@8 | 1.620E+9 | 2.025E-3 | 5.59 | 0.15 |
| Qinghu@9 | 1.614E+9 | 2.024E-3 | 5.47 | 0.21 |
| Qinghu@10 | 1.608E+9 | 2.025E-3 | 5.74 | 0.24 |
| Qinghu@11 | 1.609E+9 | 2.024E-3 | 5.50 | 0.21 |
| Qinghu@13 | 1.615E+9 | 2.024E-3 | 5.46 | 0.32 |
| Qinghu@14 | 1.610E+9 | 2.024E-3 | 5.51 | 0.23 |
| Qinghu@15 | 1.619E+9 | 2.025E-3 | 5.54 | 0.26 |
| Qinghu@16 | 1.613E+9 | 2.024E-3 | 5.51 | 0.27 |
| Qinghu@17 | 1.610E+9 | 2.025E-3 | 5.68 | 0.17 |
| Qinghu@18 | 1.611E+9 | 2.024E-3 | 5.21 | 0.20 |
| Qinghu@19 | 1.606E+9 | 2.024E-3 | 5.66 | 0.34 |
| Qinghu@20 | 1.602E+9 | 2.024E-3 | 5.63 | 0.26 |
| Qinghu@21 | 1.596E+9 | 2.024E-3 | 5.50 | 0.18 |
| Qinghu@22 | 1.591E+9 | 2.023E-3 | 5.30 | 0.24 |
| Qinghu@23 | 1.586E+9 | 2.024E-3 | 5.59 | 0.27 |

**Table S8.** Lu-Hf-Yb isotope data for the reference zircons measured via LA-MC-ICP-MS.

|  | ^177^Hf | | ^180^Hf | ^176^Yb/^177^Hf | 2SE | ^176^Lu/^177^Hf | 2SE | ^176^Hf/^177^Hf | 2SE |
| --- | --- | --- | --- | --- | --- | --- | --- | --- | --- |
| **91500** | | | | | | | | | |
| 91500-1 | | 1.627664 | 3.144839 | 0.008422 | 0.000014 | 0.000341 | 0.000000 | 0.282286 | 0.000018 |
| 91500-10 | | 1.652320 | 3.188722 | 0.008146 | 0.000013 | 0.000333 | 0.000000 | 0.282320 | 0.000020 |
| 91500-11 | | 1.629503 | 3.143976 | 0.006268 | 0.000006 | 0.000260 | 0.000000 | 0.282293 | 0.000020 |
| 91500-12 | | 1.657093 | 3.197171 | 0.007463 | 0.000006 | 0.000305 | 0.000000 | 0.282276 | 0.000018 |
| 91500-13 | | 1.664010 | 3.210257 | 0.007363 | 0.000006 | 0.000301 | 0.000000 | 0.282284 | 0.000018 |
| 91500-14 | | 1.625557 | 3.136035 | 0.005351 | 0.000074 | 0.000224 | 0.000003 | 0.282258 | 0.000017 |
| 91500-15 | | 1.560640 | 3.010937 | 0.007211 | 0.000028 | 0.000295 | 0.000001 | 0.282301 | 0.000020 |
| 91500-16 | | 1.569996 | 3.028963 | 0.006862 | 0.000033 | 0.000282 | 0.000001 | 0.282288 | 0.000019 |
| 91500-17 | | 1.595404 | 3.079235 | 0.007453 | 0.000007 | 0.000302 | 0.000000 | 0.282283 | 0.000017 |
| 91500-18 | | 1.590446 | 3.069824 | 0.007440 | 0.000008 | 0.000301 | 0.000000 | 0.282283 | 0.000020 |
| 91500-19 | | 1.579769 | 3.048773 | 0.007456 | 0.000008 | 0.000302 | 0.000000 | 0.282317 | 0.000019 |
| 91500-2 | | 1.686070 | 3.257064 | 0.006447 | 0.000093 | 0.000265 | 0.000004 | 0.282293 | 0.000016 |
| 91500-20 | | 1.566737 | 3.023602 | 0.007429 | 0.000007 | 0.000301 | 0.000000 | 0.282285 | 0.000019 |
| 91500-21 | | 1.597771 | 3.083330 | 0.007433 | 0.000008 | 0.000301 | 0.000000 | 0.282291 | 0.000019 |
| 91500-22 | | 1.592417 | 3.073255 | 0.007517 | 0.000009 | 0.000305 | 0.000000 | 0.282277 | 0.000020 |
| 91500-23 | | 1.561962 | 3.014222 | 0.007408 | 0.000007 | 0.000301 | 0.000000 | 0.282283 | 0.000016 |
| 91500-24 | | 1.568335 | 3.026431 | 0.007284 | 0.000007 | 0.000296 | 0.000000 | 0.282284 | 0.000017 |
| 91500-25 | | 1.549805 | 2.990782 | 0.007475 | 0.000006 | 0.000303 | 0.000000 | 0.282291 | 0.000020 |
| 91500-26 | | 1.572935 | 3.035692 | 0.007339 | 0.000007 | 0.000298 | 0.000000 | 0.282281 | 0.000018 |
| 91500-27 | | 1.584588 | 3.057900 | 0.007537 | 0.000009 | 0.000306 | 0.000000 | 0.282296 | 0.000018 |
| 91500-28 | | 1.568369 | 3.026822 | 0.007358 | 0.000007 | 0.000299 | 0.000000 | 0.282289 | 0.000016 |
| 91500-29 | | 1.565291 | 3.020802 | 0.007460 | 0.000007 | 0.000303 | 0.000000 | 0.282281 | 0.000019 |
| 91500-3 | | 1.695464 | 3.274521 | 0.007337 | 0.000024 | 0.000299 | 0.000001 | 0.282277 | 0.000019 |
| 91500-30 | | 1.575651 | 3.040857 | 0.007387 | 0.000008 | 0.000300 | 0.000000 | 0.282320 | 0.000019 |
| 91500-31 | | 1.572345 | 3.034601 | 0.007426 | 0.000008 | 0.000302 | 0.000000 | 0.282305 | 0.000018 |
| 91500-4 | | 1.666023 | 3.217405 | 0.005999 | 0.000007 | 0.000250 | 0.000000 | 0.282278 | 0.000017 |
| 91500-5 | | 1.649072 | 3.184230 | 0.008417 | 0.000007 | 0.000342 | 0.000000 | 0.282301 | 0.000019 |
| 91500-6 | | 1.702435 | 3.286272 | 0.007492 | 0.000006 | 0.000305 | 0.000000 | 0.282313 | 0.000018 |
| 91500-7 | | 1.664552 | 3.213144 | 0.007437 | 0.000006 | 0.000303 | 0.000000 | 0.282285 | 0.000018 |
| 91500-8 | | 1.644401 | 3.173756 | 0.007424 | 0.000007 | 0.000302 | 0.000000 | 0.282291 | 0.000017 |
| 91500-9 | | 1.645675 | 3.176084 | 0.007426 | 0.000006 | 0.000303 | 0.000000 | 0.282302 | 0.000017 |
| **Mud Tank zircon** | | | | | | | | | |
| mt-1.xls | | 2.651471 | 5.123306 | 0.000632 | 0.000004 | 0.000022 | 0.000000 | 0.282480 | 0.000013 |
| mt-10.xls | | 2.616690 | 5.049641 | 0.001496 | 0.000016 | 0.000052 | 0.000001 | 0.282508 | 0.000013 |
| mt-11.xls | | 2.538534 | 4.897644 | 0.002078 | 0.000004 | 0.000071 | 0.000000 | 0.282496 | 0.000013 |
| mt-12.xls | | 2.637911 | 5.089402 | 0.000503 | 0.000003 | 0.000018 | 0.000000 | 0.282499 | 0.000014 |
| mt-13.xls | | 2.490606 | 4.804903 | 0.001405 | 0.000016 | 0.000049 | 0.000001 | 0.282506 | 0.000014 |
| mt-14.xls | | 2.646944 | 5.106372 | 0.000433 | 0.000004 | 0.000015 | 0.000000 | 0.282498 | 0.000014 |
| mt-15.xls | | 2.452983 | 4.732356 | 0.002010 | 0.000010 | 0.000069 | 0.000000 | 0.282497 | 0.000014 |
| mt-16.xls | | 2.564885 | 4.948345 | 0.000484 | 0.000004 | 0.000018 | 0.000000 | 0.282509 | 0.000014 |
| mt-17.xls | | 2.697175 | 5.206067 | 0.000522 | 0.000003 | 0.000019 | 0.000000 | 0.282490 | 0.000013 |
| mt-18.xls | | 2.692783 | 5.197244 | 0.000387 | 0.000009 | 0.000014 | 0.000000 | 0.282479 | 0.000012 |
| mt-19.xls | | 2.724642 | 5.258769 | 0.000276 | 0.000006 | 0.000010 | 0.000000 | 0.282488 | 0.000013 |
| mt-2.xls | | 2.571912 | 4.968119 | 0.002080 | 0.000004 | 0.000071 | 0.000000 | 0.282480 | 0.000012 |
| mt-20.xls | | 2.634224 | 5.083795 | 0.000513 | 0.000003 | 0.000018 | 0.000000 | 0.282485 | 0.000015 |
| mt-21.xls | | 2.710322 | 5.230677 | 0.000258 | 0.000003 | 0.000009 | 0.000000 | 0.282478 | 0.000013 |
| mt-22.xls | | 2.543316 | 4.908427 | 0.000772 | 0.000003 | 0.000028 | 0.000000 | 0.282477 | 0.000014 |
| mt-23.xls | | 2.539606 | 4.900889 | 0.000762 | 0.000004 | 0.000028 | 0.000000 | 0.282478 | 0.000014 |
| mt-24.xls | | 2.577200 | 4.973524 | 0.000716 | 0.000005 | 0.000026 | 0.000000 | 0.282492 | 0.000014 |
| mt-25.xls | | 2.683558 | 5.178781 | 0.000276 | 0.000003 | 0.000010 | 0.000000 | 0.282491 | 0.000014 |
| mt-26.xls | | 2.549446 | 4.920114 | 0.000773 | 0.000005 | 0.000028 | 0.000000 | 0.282492 | 0.000013 |
| mt-27.xls | | 2.577465 | 4.973948 | 0.001855 | 0.000013 | 0.000064 | 0.000000 | 0.282479 | 0.000014 |
| mt-28.xls | | 2.607490 | 5.032076 | 0.000431 | 0.000011 | 0.000016 | 0.000000 | 0.282485 | 0.000012 |
| mt-29.xls | | 2.447394 | 4.722758 | 0.000694 | 0.000003 | 0.000025 | 0.000000 | 0.282486 | 0.000014 |
| mt-3.xls | | 2.725962 | 5.264711 | 0.000579 | 0.000006 | 0.000021 | 0.000000 | 0.282479 | 0.000013 |
| mt-30.xls | | 2.625273 | 5.066452 | 0.000465 | 0.000004 | 0.000017 | 0.000000 | 0.282485 | 0.000011 |
| mt-31.xls | | 2.523341 | 4.869706 | 0.000625 | 0.000003 | 0.000023 | 0.000000 | 0.282490 | 0.000013 |
| mt-4.xls | | 2.752866 | 5.316508 | 0.000423 | 0.000004 | 0.000015 | 0.000000 | 0.282494 | 0.000012 |
| mt-5.xls | | 2.753541 | 5.317002 | 0.000267 | 0.000003 | 0.000010 | 0.000000 | 0.282477 | 0.000012 |
| mt-6.xls | | 2.727296 | 5.264702 | 0.000253 | 0.000003 | 0.000009 | 0.000000 | 0.282483 | 0.000012 |
| mt-7.xls | | 2.753695 | 5.315736 | 0.000371 | 0.000004 | 0.000013 | 0.000000 | 0.282486 | 0.000013 |
| mt-8.xls | | 2.619977 | 5.057033 | 0.000770 | 0.000004 | 0.000028 | 0.000000 | 0.282492 | 0.000014 |
| mt-9.xls | | 2.601437 | 5.020727 | 0.000764 | 0.000003 | 0.000028 | 0.000000 | 0.282479 | 0.000013 |

References:

1. Chen Z, Zeng Z, Wang X, Peng X, Zhang Y, Yin X, Chen S, Zhang L, & Qi H. Element and Sr isotope zoning in plagioclase in the dacites from the southwestern Okinawa Trough: Insights into magma mixing processes and time scales. *Lithos* **376-377**, 105776 (2020).

2. Chen, Z., Zeng, Z., Yin, X., Wang, X., Zhang, Y., Chen, S., Shu, Y., Guo, K., & Li, X. Petrogenesis of highly fractionated rhyolites in the southwestern Okinawa Trough: Constraints from whole‐rock geochemistry data and Sr-Nd-Pb-O isotopes. *Geol. J.* **54**, 316-332 (2019).

3. Schmitt, A. K., Stockli, D. F., & Hausback, B. P. Eruption and magma crystallization ages of Las Tres Vírgenes (Baja California) constrained by combined ^230^Th/^238^U and (U–Th)/He dating of zircon. *J. Volcanol. Geotherm. Res.* **158**, 281–295 (2006).

4. Liu, Y., Li, X. H., Li, Q. L., Tang, G. Q., & Yin, Q. Z. Precise U-Pb zircon dating at a scale of <5 micron by the CAMECA 1280 SIMS using a Gaussian illumination probe. *J. Anal. At. Spectrom*. **26**, 845 (2011).

5. Boehnke, P., Barboni, M., & Bell, E. Zircon U/Th model ages in the presence of melt heterogeneity. *Quat. Geochronol.* **34**, 69-74 (2016).

6. Bell, E. A., & Harrison, T. M., 2013. Post-Hadean transitions in Jack Hills zircon provenance: A signal of the Late Heavy Bombardment?. *Earth Planet. Sci. Lett.* **364**, 1-11 (2013).

7. Li, X. H., Liu, Y., Li, Q. L., Guo, C. H., & Chamberlain, K. R. Precise determination of Phanerozoic zircon Pb/Pb age by multicollector SIMS without external standardization. *Geochem. Geophys. Geosyst.* **10**, Q04010 (2009).

8. Sláma, J., Košler, J., Condon, D. J., Crowley, J. L., Gerdes, A., Hanchar, J. M., Horstwood, M. S., Morris, G. A., Nasdala, L., & Norberg, N. Plešovice zircon-a new natural reference material for U-Pb and Hf isotopic microanalysis. *Chem. Geol.* **249**, 1-35 (2008).

9. Wiedenbeck, M., Alle, P., Corfu, F., Griffin, W., Meier, M., Oberli, F. v., Quadt, A. v., Roddick, J., & Spiegel, W. Three natural zircon standards for U‐Th‐Pb, Lu‐Hf, trace element and REE analyses. *Geostandards newsletter* **19**, 1-23 (1995).

10. Li, Q. L., Li, Xian-Hua, Liu, Y., Tang, G. Q., Yang, J. H., & Zhu, W. G. Precise U-Pb and Pb–Pb dating of Phanerozoic baddeleyite by SIMS with oxygen flooding technique. *J. Anal. At. Spectrom.* **25**, 1107-1113 (2010).

11. Stacey, J., & Kramers, J. Approximation of terrestrial lead isotope evolution by a two-stage model. *Earth Planet. Sci. Lett.* **26**, 207-221 (1975).

12. Ludwig, K. Users Manual for Isoplot/Ex rev. 2.49. Berkeley Geochronology Center, Berkeley. CA, Special Publications, 1a, 1, p. 56 (2001).

13. Li, X., Tang, G., Gong, B., Yang, Y., Hou, K., Hu, Z., Li, Q., Liu, Y., & Li, W. Qinghu zircon: A working reference for microbeam analysis of U-Pb age and Hf and O isotopes. *Chin. Sci. Bull.* **58**, 4647-4654 (2013).

14. Tang, G. Q., Li, X. H., Li, Q. L., Liu, Y., Ling, X. X., & Yin, Q. Z. Deciphering the physical mechanism of the topography effect for oxygen isotope measurements using a Cameca IMS-1280 SIMS. *J. Anal. At. Spectrom.* **30**, 950-956 (2015).

15. Faghihi, V., & Meijer, H. A. J., Gröning, M. A thoroughly validated spreadsheet for calculating isotopic abundances (^2^H, ^17^O, ^18^O) for mixtures of waters with different isotopic compositions. *Rapid Commun. Mass Spectrom.* **29**, 1351-1356 (2015).

16. Li, X. H., Long, W. G., Li, Q. L., Liu, Y., Zheng, Y. F., Yang, Y. H., Chamberlain, K. R., Wan, D. F., Guo, C. H., & Wang, X. C. Penglai zircon megacrysts: a potential new working reference material for microbeam determination of Hf–O isotopes and U-Pb age. *Geostand. Geoanal. Res.* **34**, 117-134 (2010).

17. Woodhead, J., Hergt, J., Shelley, M., Eggins, S., & Kemp, R. Zircon Hf-isotope analysis with an excimer laser, depth profiling, ablation of complex geometries, and concomitant age estimation. *Chem. Geol.* **209**, 121–135 (2004).

18. Woodhead, J. D., & Hergt, J. M. A preliminary appraisal of seven natural zircon reference materials for in situ Hf isotope determination. *Geostand. Geoanal. Res.* **29**, 183-195 (2005).

19. Bas, M. J., Maitre, R. W., Streckeisen, A., & Zanettin, B., A. Chemical classification of volcanic rocks based on the Total Alkali-Silica Diagram. *J. Petrol.* **27**, 745-750. (1986).

20. Roberts, M. P., & Clemens, J. D. Origin of high-potassium, calc-alkaline, I-type granitoids. *Geology* **21**, 825-828. (1993).

21. Li, X., Li, W., Wang, X., Li, Q., Liu, Y., & Tang, G. Role of mantle-derived magma in genesis of early Yanshanian granites in the Nanling Range, South China:in situzircon Hf-O isotopic constraints. *Sci.China Ser. D: Earth Sci.* **52**, 1262-1278 (2009).

22. McDonough, W. F., & Sun, S. S. The composition of the Earth. *Chem. Geol.* **120**, 223-253 (1995).

23. Griffin, W., Pearson, N J., Belousova, E., Jackson, SE v., Van Achterbergh, E, O’Reilly, Suzanne Y, & Shee, S R. The Hf isotope composition of cratonic mantle: LAM-MC-ICPMS analysis of zircon megacrysts in kimberlites. *Geochim. Cosmochim. Acta* **64**, 133-147. (2000).
